# Interleukin-6 Interweaves the Bone Marrow Microenvironment, Bone Loss, and Multiple Myeloma

**DOI:** 10.3389/fendo.2018.00788

**Published:** 2019-01-08

**Authors:** Danielle Harmer, Carolyne Falank, Michaela R. Reagan

**Affiliations:** ^1^Reagan Laboratory, Maine Medical Center Research Institute, Scarborough, ME, United States; ^2^Graduate School of Biomedical Sciences and Engineering, University of Maine, Orono, ME, United States; ^3^School of Medicine, Tufts University, Boston, MA, United States

**Keywords:** multiple myeloma, IL-6, interleukin 6, bone marrow, MSCs

## Abstract

The immune system is strongly linked to the maintenance of healthy bone. Inflammatory cytokines, specifically, are crucial to skeletal homeostasis and any dysregulation can result in detrimental health complications. Interleukins, such as interleukin 6 (IL-6), act as osteoclast differentiation modulators and as such, must be carefully monitored and regulated. IL-6 encourages osteoclastogenesis when bound to progenitors and can cause excessive osteoclastic activity and osteolysis when overly abundant. Numerous bone diseases are tied to IL-6 overexpression, including rheumatoid arthritis, osteoporosis, and bone-metastatic cancers. In the latter, IL-6 can be released with growth factors into the bone marrow microenvironment (BMM) during osteolysis from bone matrix or from cancer cells and osteoblasts in an inflammatory response to cancer cells. Thus, IL-6 helps create an ideal microenvironment for oncogenesis and metastasis. Multiple myeloma (MM) is a blood cancer that homes to the BMM and is strongly tied to overexpression of IL-6 and bone loss. The roles of IL-6 in the progression of MM are discussed in this review, including roles in bone homing, cancer-associated bone loss, disease progression and drug resistance. MM disease progression often includes the development of drug-resistant clones, and patients commonly struggle with reoccurrence. As such, therapeutics that specifically target the microenvironment, rather than the cancer itself, are ideal and IL-6, and its myriad of downstream signaling partners, are model targets. Lastly, current and potential therapeutic interventions involving IL-6 and connected signaling molecules are discussed in this review.

## Introduction

For many years, the skeletal system was regarded purely as a means for structural support and movement; however, abundant data have since been collected that reveal a complex bi-directional interaction between bone cells and surrounding bone marrow (BM) cells. BM is composed of a heterogeneous cell population including adipocytes, chondrocytes, endothelial cells, immune cells, fibroblasts, and two multipotent stem cell populations: mesenchymal stem cells (MSCs) and haematopoietic stem cells (HSCs). MSCs can differentiate into chondrocytes, bone marrow adipocytes (BMAs), and osteoblasts (bone-forming cells), while HSCs give rise to both the myeloid and lymphoid immune cell populations. The myeloid cell precursors are of particular interest as they can give rise to both innate monocyte/macrophage immune cells, but can also fuse and differentiate to form osteoclasts, the cells responsible for bone resorption.

BM cells are involved in activating and intensifying numerous signaling pathways that contribute to diseased states. The instigation of these signaling pathways can be coupled to the secretion of soluble factors by bone and stromal cells, alike. Such soluble factors include interleukin 6 (IL-6), insulin-like growth factor-1 (IGF-1), stromal cell-derived factor-1 (SDF-1), tumor necrosis factor alpha (TNF-α), interleukin-8 (IL-8), interleukin-17 (IL-17) and vascular endothelial growth factor (VEGF), which play roles in inflammation, immunosuppression, tumorigenesis, and osteolysis and thus contribute to many disease states. A number of these molecules activate NF-κB signaling, which is key in the BMM, as it binds to and activates important transcription factors and signaling cascades.

The NF-κB molecule associates with STAT3, which then induces the expression of wound healing and cancer gene products (1), such as anti-apoptotic proteins. Furthermore, STAT3 signaling results in the release of inflammatory molecules, such as IL-6 (2), SDF-1 (3), TNF-α (4), interleukin-1 (IL-1) and (IL-6) (5), which are all known to stimulate NF-κB signaling, thus creating a positive feedback loop and increased downstream inflammatory cytokine production (2). This vicious circle of NF-κB signaling can be detrimental to bone due to chronic inflammation and damage, yet it remains key in the maintenance of healthy bone, as it facilitates osteoclast differentiation and survival (6) and thus bone homeostasis.

Receptor activator of nuclear factor kappa-B ligand (RANKL) is a molecule expressed on osteoblasts which can facilitate the fusion of myeloid progenitors into osteoclasts (7). When RANKL binds to progenitor cells, it promotes NF-κB signaling, blockade of TNF receptor associated factor-3 (TRAF-3), promotion of NF-κB inducing kinase (NIK), and a consequential translocation of RelB and p52 (NF-κB2) into cell nuclei (8), which promotes signaling for osteoclastogenesis (8). MAPK pathways, particularly those involving p38, feed into this system, as they are both activated by NF-κB, but furthermore, are known to result in the production of RANKL. IL-1, for example, can stimulate RANKL via p38 in stromal cells thus promoting osteoclastogenesis (9). The extent of osteoclastogenesis is regulated by an equilibrium between RANKL and its inhibitor, osteoprotegerin (OPG).

The regulation of these bone signaling pathways is key in the limitation of bone disease. Inflammatory cytokines can disrupt ratios of RANKL:OPG and can result in excessive osteoclastogenesis. IL-6, is tied to excessive promotion of RANKL and inhibition of OPG and thus has been linked to bone osteolysis (10), osteoporosis (11), rheumatoid arthritis (11) and other bone-related pathologies. Osteolysis has further implications as it allows for the release of growth factors which promote the homing and survival of bone-metastatic cancers (12), such as prostate (13), breast (14) neuroblastoma (15), acute myeloid leukemia (AML) (16) and MM (17, 18).

MM is a blood cancer which epitomizes the crosstalk between the immune and bone systems. It is a commonly refractory, and thus recurrent, pathology characterized by uncontrollable clonal expansion of plasma cells. Myeloma cells grow in the BMM where they disrupt the delicate balance between bone growth and resorption through the secretion of factors that directly and indirectly promote osteoclasts and inhibit osteoblasts. This interaction between myeloma cells and the BMM, and the potential to activate dormant immune cells to kill myeloma cells in the BM, demonstrate exciting areas of osteoimmunology under scrutiny. It is the hope that a better understanding of the signals in the BM may allow for the identification of better targets and development of better combination therapies for MM and other bone disease. This review focuses on just one signaling pathway, IL-6, and its ability to promote both diseased and healthy states within the BMM. Since MM is strongly tied to IL-6, it will be used frequently as a disease example to emphasize the role of IL-6 in bone osteolysis, bone-metastatic cancer and general bone disease.

## IL-6 and Bone Remodeling

Healthy bone is maintained by continuous bone resorption and regrowth, which is held at equilibrium through numerous signaling cascades (19, 20). Osteoclasts secrete acidic collagenases that break down matrices and form resorption pits. Osteoblasts line the border of mineralized bone and produce and deposit collagen, osteocalcin, and osteopontin to form an osteoid matrix. Osteoblasts then calcify osteoid through deposition of calcium phosphate, calcium carbonate and hydroxyapatite. Osteocytes, or other systemic signals, control the balance of bone resorption and formation. Osteocytes, the most abundant cell type in bone, are mechanosensing cells that produce signaling molecules in response to changes in pressure, sheer stress, or BMM chemical signals. Sclerostin is a signaling molecule that is synthesized and secreted by osteocytes, and acts as a WNT inhibitor causing inhibited osteoblast differentiation and supporting adipogenesis (21). Osteocytes also secrete Dentin-Matrix-acidic-Phosphoprotein-1(DMP-1), Matrix-Extracellular-Phospho-glycoprotein (MEPE), RANKL, Fibroblast-Growth-Factor-23 (FGF-23) and Phosphate-regulating-neutral-Endopeptidase-X-linked (PHEX), all of which play roles in regulating bone mineralisation, cell fate or phosphate homeostasis (21, 22).

**Figure 1:**
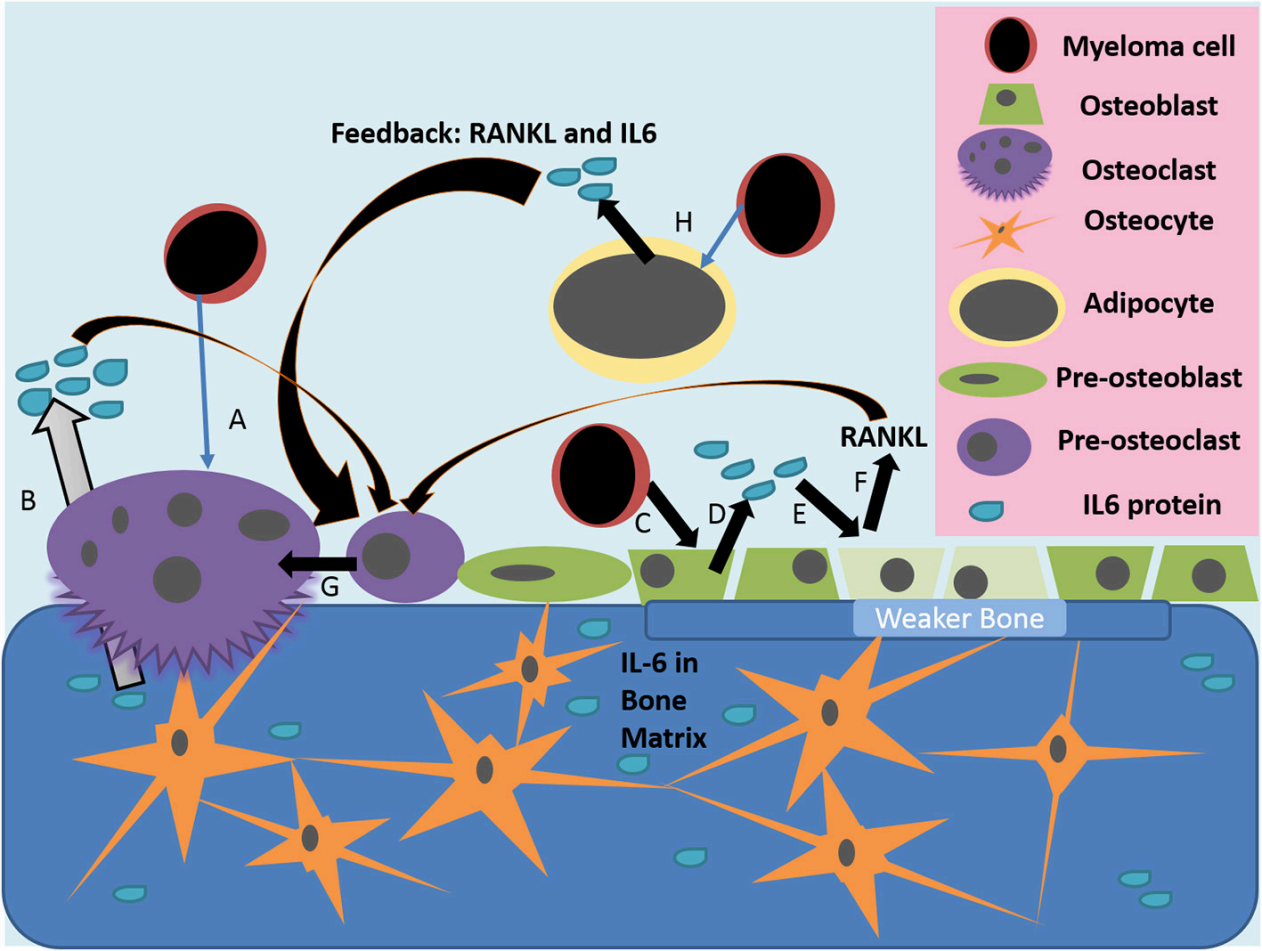
Actions of IL-6 in the diseased bone marrow microenvironment. (A) Myeloma cells or other inflammatory triggers can induce osteoclast differentiation and osteolytic activity, which releases growth factors and IL-6 stored in the bone matrix (B). (C) Myeloma cells, or other inflammatory mediators, activate the release of IL-6 from osteoblasts (D); IL-6 then inhibits the activity of osteoblasts and induces their production of RANKL (E). (F,G) IL-6 from many sources, as well as RANKL from osteoblasts, induce pre-osteoclasts to differentiate into mature osteoclasts, which then resorb bone to induce greater release of stored growth factors, creating a vicious cycle. (H) Myeloma cells also alter bone marrow adipocytes to make a more supportive niche for tumor cells and to increase osteoclastic activity through IL-6 and other molecules.

Inflammatory cytokines, such as IL-6 and IL-11, can modulate skeletal homeostasis and osteoclast differentiation. When IL-6 binds receptors on pre-osteoclasts, they promote osteoclastogenesis (23), resulting in increased levels of bone resorption (Figure 1). Along with its direct roles on osteoclastogenesis, IL-6 also alters bone remodeling; when activated by IL-6, osteoblasts induce JAK/STAT3 pathways and ultimately secrete pro-osteoclast mediators, including RANKL, IL-1, parathyroid hormone related protein (PTHrP), and prostaglandin E2 (PGE2) (12, 24–27). PGE2 and PTHrP are particularly interesting factors as both, along with parathyroid hormone (PTH) and active vitamin D [1,25(OH)2D3], have been shown to stimulate IL-6 and RANKL production in osteoblasts within the BMM (28, 29). Thus, PGE2 and PTHrP reside both upstream and downstream of IL-6 signaling, facilitating a positive feedback loop that can exacerbate the detrimental pro-osteoclastic mechanisms seen in many diseased states. IL-6 can promote PTHrP secretion via TNF-α, which itself enhances osteoclastogenesis, bone loss and hypercalcaemia (30). The pathways that use osteoblastic mediation require the soluble IL-6 receptor (sIL-6R), as osteoblasts ordinarily express only low levels of the endogenous receptor (12). Soluble IL-6R forms as a result of cleavage or alternate splicing of IL-6R and is found in circulation in low numbers due to secretion from cells (31), commonly CD4 T cells (32). Therefore, it is necessary for IL-6 to meet and bind to sIL-6R within circulation, and then once bound, fuse with the osteoblastic membrane to trigger IL-6-inducible signaling pathways. Consequently, high levels of both IL-6 and sIL-6R are markers for osteolytic disorders, such as rheumatoid arthritis (10).

IL-6 also exacerbates osteolysis through inhibition of osteoblast differentiation, further disrupting the balance of healthy bone turnover. Both IL-6 and sIL-6R can cause a decrease in osteoblastic differentiation by reducing expression of genes involved in osteoblastic differentiation including alkaline phosphatase (ALP), Runx2 and osteocalcin (33). IL-6/sIL-6R signaling can also reduce the ability of osteoblasts to mineralise bone (Figure 1). This was determined to be a result of MEK/ERK and PI3K/AKT2 pathways; inhibiting these pathways increased expression of Runx2 and other mature osteoblastic phenotypes (33). Similarly, c-Src (a proto-oncogenic tyrosine kinase) has been shown to activate STAT3 and stimulate IL-6 within immature osteoblasts (34). This stimulation can induce expression of insulin-like growth factor 5 (IGFP5)—a molecule thought to act in a paracrine fashion to inhibit osteoclastogenesis (34). Overall, IL-6 has been shown to have anti-osteogenic and pro-osteoclastic effects leading to a net inhibition of bone.

## IL-6 and Non-Cancerous Bone Disease

Regulation of IL-6 plays a crucial role in bone maintenance and remodeling. Commonly, when chronic injury and/or inflammation occurs, it instigates overexpression of IL-6 within the BMM resulting in pro-osteoclastic pathways, osteopenia, osteoporosis, and an increased fracture risk (12). In fact, diseases characterized by excessive bone loss commonly coincide with IL-6 and RANKL overproduction (10). Numerous signaling molecules interact with IL-6 to accelerate bone disease including PGE2/COX2, PTH, 1,25(OH)2D3, estrogen, and VEGF, as described below.

PGE2 results from metabolism of the fatty acid arachidonic acid via cyclooxygenase-2 (COX-2). Overexpression of COX-2 is driven by IL-6 signaling and results in increased production PGE2 (35). PGE2 interacts with G-coupled receptors EP1, EP2, and EP4, which activate tissue-specific downstream signaling pathways (36). Inflammatory conditions such as rheumatoid arthritis and osteoarthritis appear are tied PGE2-induced inflammation (37). Mast cells (38), Th17 cells (39) and dendrites (40) can all be stimulated by PGE2 via EP2 and EP4, resulting in the release of pro-inflammatory cytokines, e.g., IL-6 (41). PGE2 is also linked to osteoclastogenesis and breakdown of juxta-articular-bone and cartilage (42). PGE2 further disrupts bone homeostasis due to its ability to inhibit OPG secretion by osteoblasts (27). Use of anti-IL-6 antibodies in osteoblastic and monocyte/macrophage-like cell lines demonstrated increased OPG secretion through alleviation of PGE2-driven inhibition (27), thus concluding that PGE2 increases IL-6 signaling, driving osteoclastogenesis in part through OPG inhibition.

Similar to PGE2, PTH acts both upstream and downstream of IL-6 signaling. This hormone is expressed in response to hypocalcaemia and, once in circulation, it acts upon osteoblasts to both increase their activity and induce secretion of factors such as RANKL and IL-6 (43), which promote osteoclastic activity. PTH-induced bone loss can be inhibited by blocking IL-6R with neutralizing antibodies (44). Patients who suffer from primary parathyroidism show elevated IL-6 and markers of increased bone resorption (45), further demonstrating how this hormone is involved in IL-6-associated bone diseases.

It would be overly simple to say that IL-6 is solely detrimental to bone, as recent *in vivo* work has shown a key role for it in bone repair. Transgenic mice overexpressing IL-6 have demonstrated enhanced bone loss, faulty osteoid ossification, and reduced osteoblast activity (46), but, other studies have also shown that IL-6 knock-out (KO) mice display abnormalities in bone architecture and delayed fracture healing (47). These IL-6 KO mice demonstrated delayed mineralization and remodeling of bone, with enhanced levels of collagen and cartilage at early stages of healing and reduced osteoclast number (47). This demonstrates a necessity for IL-6-induced bone loss in the correct repair of fractures. This is further evidenced in a diabetes-associated fracture model where reduced osteoclastic activity resulted in delayed repair (48).

### IL-6 and Rheumatoid Arthritis (RA)

RA is a chronic, inflammatory condition prevalent in middle-aged people and is the leading cause of work-associated disability in the United States (49). Characterized by inflammation of the synovium of multiple joints of the body, sufferers exhibit pain and stiffness in hands, knees, wrists and feet. Progression of the disease results in detrimental damage to the joint and erosion of both the cartilage and bone. RA patients commonly express high levels of IL-6 intracellularly, which has been shown to have negative correlations clinically with bone mass density (BMD) (50). Increased vascular permeability and extravasation of fluid into synovial regions are key characteristics of RA and induce joint pain. Excess vascularization, a direct effect of IL-6-induced VEGF overexpression, enhances fluid build-up in joints (51). Antibodies against IL-6R can reduce VEGF expression in RA clinically (51).

In IL-6 KO mouse models, mice are protected against arthritis and have decreased osteoclast activity and bone loss (52). In addition, IL-6 neutralizing antibodies have been administered to mice in collagen-induced *in vivo* arthritis models, where they protected the mice from bone lesions and disease progression (53). Similarly, IL-6R antagonists have been shown to reduce osteoclastogenesis and reduce bone resorption in arthritic mouse models (54). The sIL-6R has also been shown to be of importance *in vivo*, as co-administration of mice with IL-6 and sIL-6R resulted in restoration of arthritic diseased states in IL-6 KO models (55). Having said this, some studies using male IL-6 KO mice demonstrated phenotypes of advanced osteoarthritis upon aging, complicating the story (56). Overall, most findings suggest that IL-6 induces RA initiation and progression (57).

Currently, targeting IL-6 therapeutically for RA has been successful in the clinic, although the negative side effects from this therapeutic approach should not be minimized. The U.S. Food and Drug Administration declined to approve Johnson & Johnson's RA anti-IL-6 drug called sirukumab, saying additional clinical data is needed to further evaluate its safety in September of 2017 (58). Sirukumab is a chimeric (murine-human) IgG1κ monoclonal antibody (mAb) that binds and neutralizes human IL-6. FDA panelists were concerned about an imbalance in the number of deaths in patients taking sirukumab compared with those taking a placebo, but Johnson & Johnson are continuing further development and testing of the drug (58). The most common causes of death were major heart problems, infection and cancers (58). However, Genentech's drug known as Actemra® (tocilizumab), the first humanized anti-IL-6 receptor agonist, has been FDA approved since 2010 for RA and was recently approved (November, 2018) as a prefilled autoinjector known as ACTPen^TM^ for RA and patients with giant cell arthritis (GCA), active polyarticular juvenile idiopathic arthritis (PJIA) or active systemic juvenile idiopathic arthritis (SJIA) (59). Tocilizumab can cause serious side effects however (59), and hence safer options are still desired for patients with RA and other inflammatory diseases. Still, effects of tocilizumab on bone look promising, as different clinical trials have reported that patients experienced a decrease in osteogenic inhibitor DKK1, CTX-1 bone turnover markers, and in erosions of bone in the hands, and an increase in OPG with bone histology and P1NP bone formation markers in blood (60–65).

In May of 2017, Regeneron Pharmaceutical's Kevzara® (sarilumab), another IL-6 receptor antagonist, received FDA approval for treatment of adult patients with moderately to severely active RA (66). Similarly to the IL-6/IL-6R-targeting drugs, sarilumab also increases the risk of serious side effects that may lead to hospitalization or death, largely due to the suppression of the immune system (66). These therapies would be greatly improved if IL-6 could be targeted in the tissues or areas of interest rather than systemically. Moreover, better success may be achievable using bi-specific antibodies, such as one termed MT-6194 currently in preclinical studies targeting IL-17A and IL-6R (67). Research into these and other innovative ways to target IL-6 are crucial new directions for the next generation of anti-IL-6/IL-6R therapies, but as many of the current therapies already have FDA approval, despite toxicities, it is unlikely that pharmaceutical industries feel the financial incentive to move in this direction in their Research and Development departments. It may depend on academic scientists, doctors, patients, or companies that do not have a currently FDA approved anti-IL-6 therapeutic, to push for improvements and innovations in targeting IL-6.

### IL-6 and Osteoporosis

**Figure 2:**
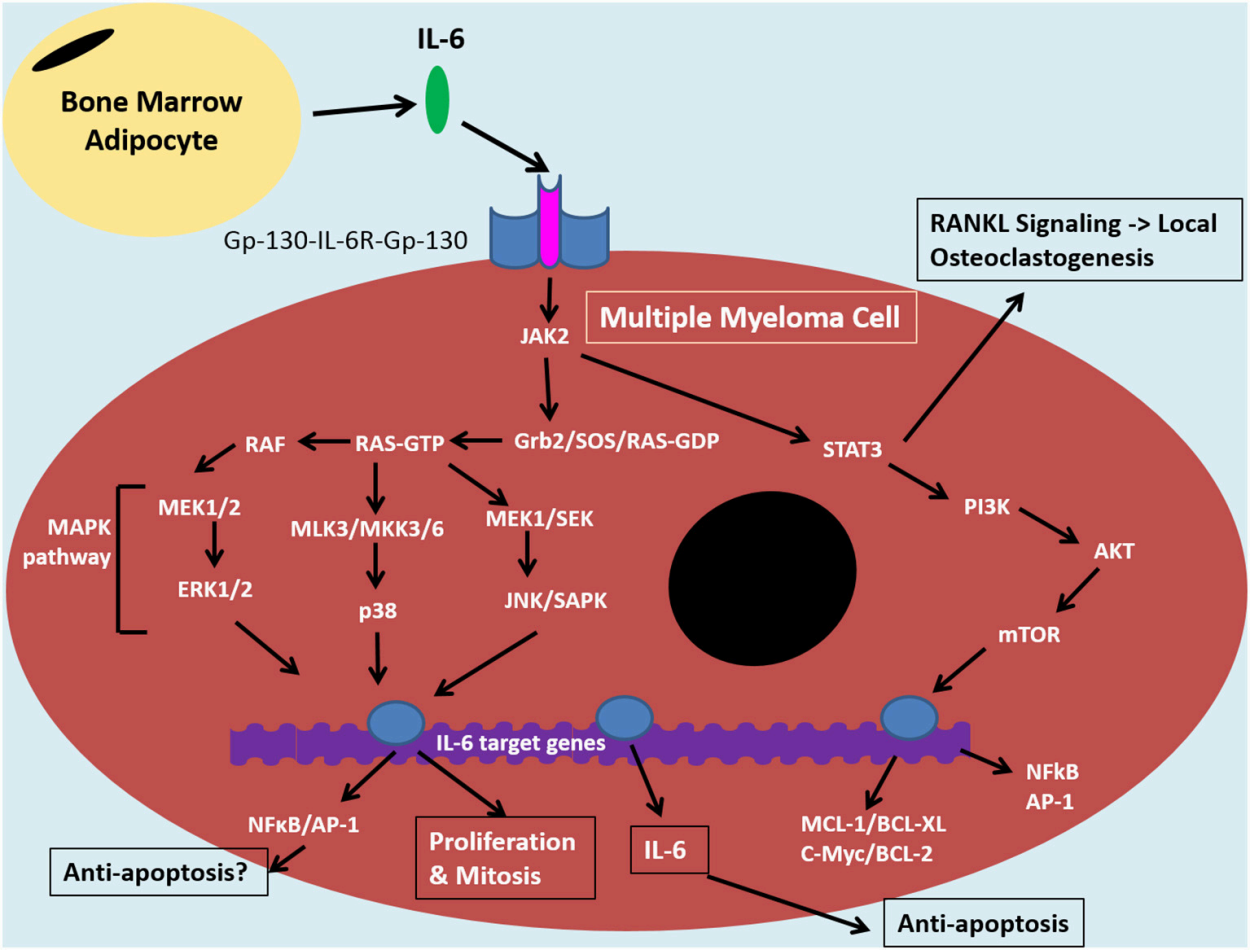
IL-6 signaling in the tumor cell of the bone marrow niche. The bone marrow microenvironment augments MAPK and PI3K/Akt pathways resulting in anti-apoptotic and NF-κB signaling in multiple myeloma cells. Binding of IL-6 to IL-6R and Gp-130 co-receptors induces JAK-2 signaling. This signaling cascade diverges down STAT3/PI3K/Akt pathways and various MAPK pathways, including MEK/ERK. The former is associated with promoting anti-apoptotic proteins: MCL1, BCL-XL, BCL-2 and c-Myc, which contribute to drug resistance. STAT3/PI3K/Akt can also promote NF-κB signaling which results in release of angiogenic and inflammatory molecules, such as IL-6. This can feed into an autocrine positive feedback loop. IκBα can inhibit NFκB through coupling and this interaction can be maintained by certan signals, such as by estradiol. The IL-6 signaling pathway in MM cells is similar to that of bone marrow stromal cells and overall it promotes an inflammatory microenvironment in the bone which results in bone loss, increased tumor burden and disease progression. Bone marrow adipocytes (BMAs) are one cell within the bone microoenvironment thought to feed into this system through secretion of IL-6. This can promote anti-apoptosis and disease progrssion through NF-κB signaling. BMAs, thus, represent an ideal target for MM therapies in order to reduce drug resistance and relapse, instead of targeting the complicated, clonally expanding plasma cell.

Aging increases the production of inflammatory molecules, including IL-6, which explains some of the increased prevalence of inflammatory disorders (diabetes, lupus, and RA) in aging (68–70). Osteoporosis is also common in older and post-menopausal women due to a natural reduction in estrogen levels and high IL-6 levels, adding to increased osteoclastogenesis and pronounced osteopenia (71, 72). Ordinarily, the estrogen isomer 17-β-oestradiol (E2) reduces monocyte secretion of IL-6 and IL-8 (73), and thus after menopause, these pro-osteoclastic cytokines become more abundant (74). E2 interacts with the NFκB pathway, which is ordinarily held in an inactive state by coupling with an inhibitor known as IκBα (Figure 2). Certain signals result in IκBα becoming phosphorylated, uncoupling from NFκB and releasing it from its inactive state, allowing for signaling of downstream genes (75). Studies show that E2 promotes IκBα coupling to NFκB, keeping it in an inactivate state (76). This indicates how a loss in estrogen can result in increased IL-6 and NFκB signaling, and a consequential increase in bone loss and osteoporosis.

Bone loss induced by the E2-NFκB-IL-6 pathways can be reversed by administration of E2 (72) or anti-IL-6 neutralizing antibody (77–79). Clinically, post-menopausal women demonstrate an increased risk of osteoporosis, and patients with certain polymorphisms in the IL-6 promoter show further increases in bone resorption rates and reduction in BMD (80). This highlights how variation in IL-6 expression within patients can alter the risk of osteoporosis in estrogen-depleted environments. Currently, targeting IL-6 therapeutically for osteoporosis has not been explored, but this is an interesting clinical future direction that holds great potential if the side effects from these types of therapies can be minimized.

## IL-6 and Bone-Metastatic Cancer

Paul Ehrlich first proposed the function of the immune system as the first line of defense against cancer in the early twentieth century. Since then, the roles of immune and stromal cells, and their secretory repertoires, have been extensively studied in conjunction with cancer. Inflammation in particular has been shown to be key in promoting cancer proliferation and differentiation, with IL-6 being an integral player (81). Many cancer types have been shown to be associated with high serum IL-6 including colorectal (82), breast (83), and prostate cancer (84), as well as MM (85). These cancers induce phenotypic changes in the surrounding stromal cells, creating unique cell types, such as tumor-associated macrophage (TAMs) (86, 87) and cancer-associated fibroblasts (CAFs) (76, 77). These activated stromal cells, along with CD4^+^ T cells and myeloid-derived suppressor cells (MDSCs), are the main sources of IL-6 in most tumor microenvironments, alongside the tumor cells themselves (88–92). Consequently, these BMM cells are associated with creating pro-oncogenic environments that promote proliferation and progression of cancer cells by favoring angiogenesis and metastasis, and inhibiting apoptosis (93–96). For example, knockdown of IL-6 by RNAi within CAFs can attenuate metastatic phenotypes *in vivo* (94). It is not surprising that level of IL-6 within the serum of various cancer patients is a key prognostic indicator for their response or disease progression (88). As a rule, high levels of IL-6 are associated with aggressive forms of cancers (97–100) and IL-6 levels can act as independent markers of prognosis in certain cancers (101). Furthermore, high expression of IL-6 has been more closely linked to recurrent tumors than primary tumors (102). IL-6 promotes proliferation (103), facilitates an epithelial-to-mesenchymal transition (94, 104) and enhances angiogenesis via VEGF stimulation (105, 106). Increased IL-6 in cancer cells results in subsequent release of IL-6 by stromal cells, thus feeding into the pro-tumoural feed-forward loop and exacerbating the diseased state (107–109).

Chemotherapeutics and ionizing radiation are the most common cancer therapies that work by disrupting DNA and protein synthesis pathways resulting in apoptosis. Yet both are known to stimulate oxidative stress, the NFκB pathway, and consequently IL-6 (110–114). IL-6 stimulation then counteracts the chemotherapy effect by promoting anti-apoptotic pathways to cause drug resistance (115). The anti-myeloma drug bortezomib targets the 26S subunit of the proteasome, the cellular machinery involved in degrading misfolded proteins, and causes an accumulation of unwanted/toxic proteins within cells (116). This accumulation of proteins is particularly seen in cells that already have dysfunctional or excessive protein-folding processes, such as myeloma cells (117). Misfolded protein accumulation results in increased ER stress, cell cycle arrest and programmed cell death (118). However, IL-6 has been shown to induce anti-apoptotic pathways in certain cancers, thus attenuating the efficacy of this drug (119–121). Gemcitabine is a nucleoside analog that causes termination of DNA synthesis (122). Gemcitabine targets fast-replicating cancer cells and resistance to this drug may also be tied to IL-6, as evidenced by a study where gemcitabine-resistance was observed in cancer cells treated with IL-6 and sIL-6R. This was thought to be due to a IL-6-prompted barricade of cells in G0/G1 cell cycle stages, preventing cells from entering the necessary cell-division stages necessary drug-induced replicative-associated destruction (26, 123–125).

As stated above, IL-6 also plays roles in apoptosis, which occurs due to cellular mishaps as a means to reduce malignancy. Interestingly, IL-6 has been shown to play both pro- and anti-apoptotic roles depending on tumor type. In some p53-wild-type breast and colon cancers, when tumors were treated with kinase inhibitors and general chemotherapies, IL-6 was shown to induce apoptosis via p53 and STAT5, with downstream signaling occurring through BAX [the reciprocal protein to anti-apoptotic BCL-2 (126)]. On the other hand, certain cancers, including many bone-metastases, have an opposing phenotype and demonstrate a sustained and even enhanced proliferation due to IL-6 and similar cytokines (113–115).

Bone metastatic cancer cells can become dormant within the BMM, thus protecting them from elimination by the immune system (127). It is common for these tumors to eventually become proliferative and induce osteoclastogenesis through secretion of soluble factors (128, 129). Melanoma cell lines have been shown to home to the bone and induce bone destruction through a TGF-β-IL-11 axis via osteoblasts (130), whilst other cancers facilitate similar pathways, such as TGF-β-RANKL-mediated destruction (131). IL-6 and its bone destructive roles can be tied to certain cancers, including MM and neuroblastoma, both of which induce IL-6 secretion from the surrounding stroma resulting in bone loss through the NFκB pathway (132, 133). MM cells derive from the immune plasma cell, home to the BM, cause bone destruction, and eventually become drug resistant. IL-6 plays a role in all these steps, so this review will focus on the effects of IL-6 in MM, as a disease model, from here on.

### IL-6 and MM Homing to the Bone Marrow Niche

Chemokine gradients, such as that created by SDF-1, play a large role homing of cells to the BMM. The CXCR4-SDF1 axis is known to promote IL-6 expression in numerous cancers, including MM (3, 134), and other studies have also indicated that IL-6 may promote CXCR7-SDF1 or CXCR4-SDF1 signaling (135), and as such promote bone-homing (136). SDF1 is constitutively expressed by stromal cells and osteoblasts in the BMM (127, 128), and attracts CXCR4^+^/CXCR7^+^ haemopoietic or tumor cells to the BMM (137). B-cells, MM cells, and other bone metastatic cancer cells, including breast and melanoma cells, express CXCR4, which functions in the proliferation and migration of these cells to the SDF1-rich BMM (138–141). The CXCR4-SDF1 axis can also promote the expression of molecules that enable adhesion of cells to the endothelial lining of the BM sinus, such as VCAM1 (142). Additionally, α4β1 integrins can overexpressed due to CXCR4-SDF1 signaling, which can further enhance homing to the BM (142, 143). In contrast, TGF-β1 supresses expression of SDF-1 and has been shown to reduce BM stromal cell (BMSC) migration and adhesion, which could translate to a reduced ability to traffic to the BM (144). It is unsurprising that a CXCR4 inhibitor has been shown to disrupt MM cell interaction with the BMM and thus increase sensitivity to treatment (145).

Myeloma cells also commonly express cell surface receptors CCR1 and CCR2 (146, 147), which bind MIP-1α and MCP1-3, respectively. Similar to SDF1, these ligands are expressed highly by BMSCs and promote myeloma cell migration and homing to the BMM (148, 149). All three receptor axes (CCR1, CCR2, CXCR4) are interesting from a clinical point of view as patients with low expression of these receptors have shown poor disease prognosis, associated with high serum β2 microglobulin and C-reactive protein (146). The former is a molecule secreted by B cells and acts as a marker of disease progression and poor prognosis in MM. The latter is a marker of systemic inflammation and again marks poor clinical prognosis in MM. It is possible that the downregulation of the receptors results in a reduced ability to home to and reside within the BMM and an increase in circulating myeloma cells, which may cause more systemic spreading of the disease. However, this contradicts our typical understanding that tumor cells are more protected in the BM, and thus cause worse outcomes when lodged in the bone rather than when in circulation. Interestingly, IL-6 KO mice are known to express lower levels of IL-2 (150), a cytokine known to promote CCR1 and CCR2 (151). Taken together, these data indicate that IL-6 may increase MM bone homing through promoting not only CXCR4 and possibly CCR2/3 signaling.

### IL-6–Driven Bone Destruction in MM

MM also increases osteoclast number and activity, which leads to hypercalcaemia, renal-insufficiency, anemia, osteopenia and lesions in the bone. The breakdown of bone promotes chronic pain, increases fracture risk, and results in the release of bone-derived growth factors, which feed the tumor cells and promote disease progression (17, 18). This vicious cycle leads to a poor quality of life and ultimately results in prolific dissemination of these plasma cells throughout the body, a fatal condition known as plasma cell leukemia.

A number of factors feed into MM-bone loss including osteocyte-derived sclerostin. In MM patients, elevated circulating sclerostin levels are commonly seen, particularly in advanced stages (152). Sclerostin has anti-osteoblastic properties and anti-sclerostin therapeutics are currently being investigated for use in MM-associated bone loss pre-clinically (153, 154). Anti-sclerostin antibodies also reduce BM adiposity, and hence more research into their effects on bone or cancer though modulating BM adiposity is warranted (155, 156). DKK1 is another Wnt inhibitor involved in MM-mediated bone loss and disease progression that has been investigated as a target in MM bone loss (157). DKK1, like sclerostin, inhibits osteoblastogenesis by blocking differentiation of pre-osteoblasts by impeding the WNT signaling cascade (158). Recently, a bispecific antibody targeting sclerostin and DKK-1 has been shown to promote bone mass accrual and fracture repair in rodents and non-human primates (159). In addition to simply reducing numbers of mature osteoblasts, DKK1 also increases the number of undifferentiated BMSCs, and these undifferentiated BMSCs secrete higher IL-6 amounts than their differentiated counterparts (160), thus feeding into the IL-6-bone-destruction pathway described previously. Inhibition of IL-6 from MSC-conditioned media slowed the proliferation of DKK-1 secreting myeloma cells treated with this media, suggesting that in the myeloma environment, IL-6 increases bone resorption through promoting proliferation of DKK-1-secreting myeloma cells (160). However, in other contexts, a negative correlation between IL-6 and DKK1 was observed, implying that more research into the role of IL-6 in bone destruction would be useful (161).

IL-6 mediated bone loss also occurs due to direct expression and release of IL-6 by MM cells and consequential osteoclast activation. The mechanism of IL-6-mediated osteoclastogenesis is thought to be due primarily to activation of the JAK2/STAT3 axis, resulting in upregulation of RANKL molecules (24) (see Figure 2). This overthrows the fine balance between osteoclastic RANKL molecules and their inhibitor, OPG, pushing the equilibrium in favor of bone loss (162). In addition, IL-6 may also make preosteoclasts more sensitive to RANKL stimulation, presumably due to an IL-6-mediated upregulation of RANK receptors, which was previously shown in Paget's disease (163). *In vivo* studies have confirmed IL-6 mediated bone loss in both trabecular and endochondral bone; this bone loss is enhanced in the presence of MM cells and is associated with increased osteoclast differentiation (164). This finding is being investigated in phase II clinical trials, which are currently examining the efficacy of anti-IL-6 treatment on MM bone-loss (NCT01484275).

### IL-6 and MM Disease Progression

MM begins as a condition known as monoclonal gammopathy of undetermined significance (MGUS), an asymptomatic stage where levels of immunoglobulin in the blood and risk of fracture are elevated. Blood serum IL-6 levels correspond to prognosis and progression of MM (85); prognosis is worse when levels exceed 7 pg/ml, with an average survival of 2.7 months compared to 53.7 months in those with lower levels (85). IL-6/STAT3 pathways are known to promote angiogenesis via enhancement of VEGF in MM (165). Osteoclasts are also known to secrete pro-angiogenic molecules, and thus in MM when both osteoclast number and activity is enhanced, there is also an increase in the development of vasculature (166). IL-6/STAT3 signaling not only promotes the creation of these new endothelial cells, but also stimulates Ras, Akt and MAPK pathways which promotes the survival of said MM cells (167).

### IL-6–Mediated Drug Resistance and Survival in MM

Apoptosis involves the activation of caspase enzymes in a cascade ending in the activation and release of apoptosis-inducing factors from mitochondria. Apoptosis-inducing agents cause DNA fragmentation and chromatin condensation, which ultimately induces cell death (168). IL-6 promotes JAK/STAT3 and consequently PI3K/Akt and MEK/MAPK (169), (Figure 2); these pathways are known to upregulate anti-apoptosis proteins Mcl-1, Bcl-XL and c-Myc in primary MM cells, resulting in cell survival and chemotherapy resistance (170, 171). A number of antibodies have been investigated to neutralize IL-6, but little success has occurred clinically (172, 173). However, evidence indicates that indirect targeting of downstream anti-apoptosis mediators can help reverse the effects of IL-6. *In vitro*, anti-sense targeting strategies against MCL-1 sensitized MM cells to drugs by inhibiting these anti-apoptotic pathways (174). In addition, IL-6-mediated resistance was reversed by targeting CDC34 (175). This molecule is an ubiquitin-conjugating enzyme involved in proteasomal degradation. It has been tied to ubiquitination and degradation of IκBα, the NFκB inhibitor, and thus promotes drug resistance via NFκB signaling. Targeting other molecules within the JAK/STAT3 cascade also appears to aid in resistance reversal, as the Akt inhibitor, MK2206, has also been found to help overcome bortezomib resistance in MM cells induced by co-culturing with IL-6 or MSCs (176). [For more on IL-6 and proteasome inhibitor resistance in MM, we refer the reader to our recent review may (121)].

IL-6 may also cause drug resistance through epigenetic modulation proteins. IL-6 signals via STAT3 and enhances DNA methyltransferase 1, which promotes the methylation and thus deactivation of P53, facilitating cells to avoid cell cycle checkpoint destruction (177). This could clearly have unfavorable effects in MM, promoting drug resistance and/or disease progression. Initial studies into use of general histone deacetylase (HDAC) inhibitors were unfavorable (178), however, subsequent use of selective HDAC inhibitors, such as chidamide, have shown anti-MM and bone protective effect, showing synergistic effects with other therapies such as dexamethasone, carfilzomib and pomalidomide (179).

### Bone Marrow Adipocyte (BMA)-Derived IL-6 Contribution to MM Progression

Obesity is an increasingly common condition which increases one's risk of cardiovascular disease, diabetes, certain cancers, and many other diseases. One link to cancer is due to the increased inflammatory state which occurs due to obesity (180). The excess storage of lipids within adipose tissues causes the release of inflammatory molecules, TNF-α and IL-6, and suppression of anti-inflammatory adiponectin (181). This creates a microenvironment of high oxidative stress and inflammation and results in damage to tissues, increasing one's risk of oncogenic mutations and tumorigenesis (180). Increased fat intake, which typically causes obesity over time, is linked to higher levels of BM adiposity in rodents (182); recently, BM adipocytes (BMAs) have been shown to play significant roles in MM drug resistance and disease progression (183), possibly through the secretion of soluble factors, such as IL-6 (184). BMAs have such a profound effect on MM that, clinically, obese and older populations, both of whom suffer from enhanced systemic inflammation and increased BMA levels, demonstrate higher risk of developing MM than other groups of people (185).

BMAs may contribute to disease progression in numerous ways. *In vitro* work has demonstrated an ability of BMAs extracted from MM patient femurs to support MM cell growth and protect them from chemotherapy-induced apoptosis (186). In addition, MM is known to increase expression of PGC-1α within BMAs, resulting in VEGF and GLUT-4 expression (187), which increases proliferation, angiogenesis and metastasis, as described above. BMAs have also been shown to increase expression of autophagic proteins within MM cells, which can promote drug resistance (186). Finally, BMA-derived IL-6 has been hypothesized to contribute to chemotherapy resistance due to an upregulation of anti-apoptotic proteins and inhibition of cell checkpoint proteins (Figure 2). The latter pathway remains unverified, yet studies are currently underway to investigate this further. If true, IL-6 and its downstream signaling molecules represent good targets for possible re-sensitization of MM cells in refractory disease. Investigation into the blockade of anti-apoptotic pathways downstream of IL-6 and other IL-6 mediated pathways is warranted. In addition, efforts should be made to find therapeutics that target BMAs specifically, and to investigate the secretory repertoires of all BMM cells to better understand and manipulate the microenvironment into one of anti-tumorigenicity.

### Clinically Targeting IL-6 in Cancer

Despite extensive pre-clinical *in vitro* and *in vivo* support for the role of IL-6 in MM and other osteolytic bone cancers, clinical translation results have been dismal. Clinical trial results with siltuximab, formerly CNTO 328, in MM have been disheartening and no clinical trials in other bone cancers have been initiated as of yet. As presented in an abstract for the American Society of Hematology (ASH), 2017 annual meeting, IL-6 blockade did not add benefit to chemotherapy in a phase 2, randomized, double-blind, placebo-controlled multicenter study in patients with high-risk smoldering MM (188). A clinical trial examining if siltuximab can decrease symptom burden after autologous stem cell transplantation for patients with MM or AL amyloidosis is currently underway (ClinicalTrials.gov Identifier: NCT03315026). No data on MM and siltuximab was presented at the ASH 2018 conference. A study of CNTO 328 in Japan from 2011 to 2014 in relapsed or refractory MM patients was halted due to safety concerns (ClinicalTrials.gov Identifier: NCT01309412). An open-label, phase I trial of CTNO with lenalidomide, bortezomib and dexamethasone (RVD) was performed (ClinicalTrials.gov Identifier: NCT01531998), however the efficacy data were limited by the small number of patients since the trial was halted and did not proceed to phase II. The abandonment of the phase II trial was due to the negative outcomes from another phase II study of bortezomib-melphalan-prednisone (VMP) vs. VMP+siltuximab, which demonstrated no significant improvement in progression-free survival. After this finding, further development of siltuximab in symptomatic myeloma was halted by the sponsor (189). Trials have also been initiated examining the safety and efficacy of siltuximab in metastatic, hormone-refractory prostate cancer, renal and other solid tumors (ovarian, pancreatic, colorectal, and others), and the field is currently waiting to see the results of these trials (190).

Tocilizumab, a humanized anti-IL-6R mAb, is currently the subject of investigation in many clinical cancer trials. Current clinical trials are ongoing for metastatic HER2-positive breast cancer resistant to trastuzumab, lymphoblastic leukemia, pancreatic cancer, myeloid leukemia, B-cell chronic lymphocytic leukemia, non-Hodgkin's lymphoma, B-cell lymphoma, and diffuse large B-cell lymphoma (ClinicalTrials.gov). Trials in myeloma were terminated due to low accrual rate (ClinicalTrials.gov Identifier: NCT02057770). In Europe, there is currently a clinical trial to explore the use of tocilizumab in fibrous dysplasia of bone (FD), a rare, painful bone disease affecting one or several bones (191). If promising data result, more clinical trials may arise studying the drug for bone pain relief applications.

Unfortunately, overall, large randomized trials show no efficacy of IL-6 inhibitors in various cancers, particularly myeloma. These results are despite a full inhibition of C-reactive protein (CRP) production in treated patients, the numerous preclinical studies showing an involvement of IL-6 in these diseases, and initial short-term treatments demonstrating a dramatic inhibition of cancer cell proliferation *in vivo* (192). Similarly, a meta-review of 48 clinical studies concluded that inhibition of IL-6 has unknown and unproven effects on decreasing GI (gastric, pancreatic, colorectal, bile duct and gall bladder) cancer syndromes or improving quality of life (191). A likely explanation for this lack of efficacy is the plasticity of cancer cells and their ability to clonally evolve and develop subclones that are less dependent on IL-6. Moreover, many therapies targeting tumor cells already decrease IL-6 or pathways downstream of IL-6, so that no additive or synergistic effect is derived from the anti-IL-6 therapy. Still, anti-IL-6 therapeutics are able to neutralize IL-6 production *in vivo* and are safe and useful in inflammatory diseases and Castleman disease. Their application may hold promise in treatment of bone-resident cancers if more developed (e.g., bi-specific mAbs against tumor cells and IL-6, or against RANKL and IL-6) therapies can be developed to increase their efficacy. Moreover, getting the treatment more specifically to the cancer may allow for higher doses and less off-target effects, and thus better outcomes, and more research into tumor-homing drug delivery or targeted nanomedicine technology may accelerate this process. As we understand more about the role of the immune system in cancer and the ability for tumor cells to block the immune response, researchers and clinicians may be able to design anti-IL-6 clinical trials using patient populations that are identified to have a positive response to these therapies. Similarly, a better understanding of the full activity of anti-IL-6 therapy will mean that different combination regimens or dosing strategies may be designed to be optimal for different patient subgroups.

## Conclusions

IL-6 is a pro-inflammatory cytokine that promotes NF-κB, MAPK and PI3K/Akt signaling. Together these pathways promote anti-apoptosis signaling and drug resistance in cancer cells, in addition to further inflammatory signaling. The latter contributes to bone destruction and osteopenia, which promotes homing of metastatic cancers to the bone marrow niche. Bone marrow adipocytes, as well as mesenchymal stromal cells, may be a source of IL-6 or other factors, that contribute to chemotherapy resistance in the bone microenvironment. Targeting of these adipocytes, or their secreted factors, may help alleviate refractory disease.

Because MM demonstrates such genetic heterogeneity and high levels of refractory disease, targeting the BMM, along with the tumor cell directly, is an ideal plan of attack. The IL-6 signaling pathway in MM cells promotes an inflammatory bone microenvironment that results in osteopenia, increased tumor burden and disease progression. Bone marrow adipocytes are one cell type within the bone microoenvironment thought to feed into this system through secretion of IL-6 and other adipokines. Thus, bone marrow adipocytes represent an ideal target for MM therapies in order to reduce drug resistance and relapse, instead of targeting the complicated, clonally expanding plasma cells. The more we learn, the better we can target such pathways and not only improve quality of life for patients, but hopefully extend that lifetime as well. As this dream has not become realized in the clinic yet, more efforts on understanding and maximizing the targeting of IL-6 and its downstream pathways may be necessary. Moreover, building mouse cancer models, or tissue-engineered 3D models, that more accurately model the effects of targeting IL-6 in the human should lead to better clinical results in the future and avoid the pitfalls of wasted time and money on unsuccessful clinical trials.

## Author Contributions

DH wrote the manuscript. CF and MR edited the manuscript. All authors approved the manuscript. MR takes responsibility for the manuscript.

### Conflict of Interest Statement

The authors declare that the research was conducted in the absence of any commercial or financial relationships that could be construed as a potential conflict of interest.

## References

[1] Dauer DJ Ferraro B Song L Yu B Mora L Buettner R. Stat3 regulates genes common to both wound healing and cancer. Oncogene (2005) 24:3397. 10.1038/sj.onc.1208469 15735721

[2] Baeuerle PA Henkel T Function and activation of NF-kappaB in the immune system. Annu Rev Immunol. (1994) 12:141–79. 10.1146/annurev.iy.12.040194.001041 8011280

[3] Hideshima T Chauhan D Hayashi T Podar K Akiyama M Gupta D. The biological sequelae of stromal cell-derived factor-1α in multiple myeloma. Mol Cancer Ther. (2002) 1:539–44. 12479272

[4] Hideshima T Chauhan D Schlossman R Richardson P Anderson KC. The role of tumor necrosis factor α in the pathophysiology of human multiple myeloma: therapeutic applications. Oncogene (2001) 20:4519. 10.1038/sj.onc.1204623 11494147

[5] Betts JC Cheshire JK Akirav S Kishimotov T Woo P The Role of NF-KB and NF-IL6 transactivating factors in the synergistic activation of human serum amyloid A gene expression by interleukin- 1 and interleukin-6. J Biol Chem. (1993) 268:25624–31. 8244997

[6] Xing L Bushnell TP Carlson L Tai Z Tondravi M Siebenlist U NF-κB p50 and p52 expression is not required for RANK-expressing osteoclast progenitor formation but is essential for RANK- and cytokine-mediated osteoclastogenesis. J Bone Miner Res. (2009) 17:1200–10. 10.1359/jbmr.2002.17.7.1200 12096833

[7] Arai F Miyamoto T Ohneda O Inada T Sudo T Brasel K. Commitment and differentiation of osteoclast precursor cells by the sequential expression of C-Fms and receptor activator of nuclear factor κb (Rank) receptors. J Exp Med. (1999) 190:1741–54. 10.1084/jem.190.12.1741 10601350 PMC2195707

[8] Franzoso G Carlson L Xing L Poljak L Shores EW Brown KD. Requirement for NF-κB in osteoclast and B-cell development. Genes Dev. (1997) 11:3482–96. 9407039 10.1101/gad.11.24.3482 PMC316809

[9] Wei S Kitaura H Zhou P Ross FP Teitelbaum SL. IL-1 mediates TNF-induced osteoclastogenesis. J Clin Invest. (2005) 115:282–90. 10.1172/JCI200523394 15668736 PMC544608

[10] Steeve KT Marc P Sandrine T Dominique H Yannick F IL-6, RANKL, TNF-alpha/IL-1: interrelations in bone resorption pathophysiology. Cytokine Growth Factor Rev. (2004) 15:49–60. 10.1016/j.cytogfr.2003.10.005 14746813

[11] Hashizume M Mihara M. The roles of interleukin-6 in the pathogenesis of rheumatoid arthritis. Arthritis (2011) 2011:765624. 10.1155/2011/765624 22046525 PMC3199948

[12] Tawara K Oxford JT Jorcyk CL. Clinical significance of interleukin (IL)-6 in cancer metastasis to bone: potential of anti-IL-6 therapies. Cancer Manag Res. (2011) 3:177–189. 10.2147/CMR.S18101 21625400 PMC3101113

[13] George DJ Halabi S Shepard TF Sanford B Vogelzang NJ Small EJ. The prognostic significance of plasma interleukin-6 levels in patients with metastatic hormone-refractory prostate cancer: results from cancer and leukemia group B 9480. Clin Cancer Res. (2005) 11:1815–20. 10.1158/1078-0432.CCR-04-1560 15756004

[14] Coleman RE Rubens RD. The clinical course of bone metastases from breast cancer. Br J Cancer (1987) 55:61–6. 10.1038/bjc.1987.13 3814476 PMC2001575

[15] Ara T Song L Shimada H Keshelava N Russell HV Metelitsa LS. Interleukin-6 in the bone marrow microenvironment promotes the growth and survival of neuroblastoma cells. Cancer Res. (2009) 69:329–37. 10.1158/0008-5472.CAN-08-0613 19118018 PMC2761219

[16] Stevens AM Miller JM Munoz JO Gaikwad AS Redell MS. Interleukin-6 levels predict event-free survival in pediatric AML and suggest a mechanism of chemotherapy resistance. Blood Adv. (2017) 1:1387–97. 10.1182/bloodadvances.2017007856 29296780 PMC5727855

[17] Hallek M Bergsagel PL Anderson KC. Multiple myeloma: increasing evidence for a multistep transformation process. Blood (1998) 91:3–21. 9414264 PMC3901996

[18] Kawano M Hirano T Matsuda T Taga T Horii Y Iwato K. Autocrine generation and requirement of BSF-2/IL-6 for human multiple myelomas. Nature (1988) 332:83–5. 10.1038/332083a0 3258060

[19] Hayrapetyan A Jansen JA van den Beucken JJJP. Signaling pathways involved in osteogenesis and their application for bone regenerative medicine. Tissue Eng Part B Rev. (2014) 21:75–87. 10.1089/ten.teb.2014.0119 25015093

[20] Thompson WR Rubin CT Rubin J. Mechanical regulation of signaling pathways in bone. Gene (2012) 503:179–193. 10.1016/j.gene.2012.04.076 22575727 PMC3371109

[21] McDonald MM Fairfield H Falank C Reagan MR. Adipose, bone, and myeloma: contributions from the microenvironment. Calcif Tissue Int. (2017) 100:433–48. 10.1007/s00223-016-0162-2 27343063 PMC5396178

[22] Dallas SL Prideaux M Bonewald LF The osteocyte: an endocrine cell … and more. Endocr Rev. (2013) 34:658–90. 10.1210/er.2012-1026 23612223 PMC3785641

[23] Udagawa N Takahashi N Katagiri T Tamura T Wada S Findlay D Interleukin (IL)-6 induction of osteoclast differentiation depends on IL-6 receptors expressed on osteoblastic cells but not on osteoclast progenitors. J Exp Med. (1995) 182:1461–8. 10.1084/jem.182.5.1461 7595216 PMC2192181

[24] Hashizume M Hayakawa N Mihara M. IL-6 trans-signalling directly induces RANKL on fibroblast-like synovial cells and is involved in RANKL induction by TNF-α and IL-17. Rheumatology (2008) 47:1635–40. 10.1093/rheumatology/ken363 18786965

[25] O'Brien CA Gubrij I Lin SC Saylors RL Manolagas SC STAT3 activation in stromal/osteoblastic cells is required for induction of the receptor activator of NF-κB ligand and stimulation of osteoclastogenesis by gp130-utilizing cytokines or interleukin-1 but not 1,25-dihydroxyvitamin D3 or parathyroid hormone. J Biol Chem. (1999) 274:19301–8. 10383440 10.1074/jbc.274.27.19301

[26] Blanchard F Duplomb L Baud'huin M Brounais B. The dual role of IL-6-type cytokines on bone remodeling and bone tumors. Cytokine Growth Factor Rev. (2009) 20:19–28. 10.1016/j.cytogfr.2008.11.004 19038573

[27] Liu X-H Kirschenbaum A Yao S Levine AC Cross-Talk between the interleukin-6 and prostaglandin E2 signaling systems results in enhancement of osteoclastogenesis through effects on the osteoprotegerin/Receptor Activator of Nuclear Factor-κB (RANK) ligand/RANK system. Endocrinology (2005) 146:1991–8. 10.1210/en.2004-1167 15618359

[28] Kozawa O Suzuki A Tokuda H Kaida T Uematsu T. Interleukin-6 synthesis induced by prostaglandin E2: cross-talk regulation by protein kinase C. Bone (1998) 22:355–60. 10.1016/S8756-3282(97)00293-7 9556135

[29] Gruber R Nothegger G Ho G-M Willheim M Peterlik M Differential stimulation by PGE2 and calcemic hormones of IL-6 in stromal/osteoblastic cells. Biochem Biophys Res Commun. (2000) 270:1080–5. 10.1006/bbrc.2000.2573 10772953

[30] Devlin RD Reddy SV Savino R Ciliberto G Roodman GD. IL-6 mediates the effects of IL-1 or TNF, but Not PTHrP or 1,25(OH)2D3, on osteoclast-like cell formation in normal human bone marrow cultures. J Bone Miner Res. (1998) 13:393–9. 9525339 10.1359/jbmr.1998.13.3.393

[31] Csilla Holub M Szalai C Polgár A Tóth S Falus A Generation of ‘truncated' interleukin-6 receptor (IL-6R) mRNA by alternative splicing; a possible source of soluble IL-6R. Immunol Lett. (1999) 68:121–4. 10397166 10.1016/s0165-2478(99)00040-1

[32] Briso EM Dienz O Rincon M. Soluble IL-6R is produced by IL-6R ectodomain shedding in activated CD4 T cells. J Immunol. (2008) 180:7102–6. 10.4049/jimmunol.180.11.7102 18490707 PMC2692633

[33] Kaneshiro S Ebina K Shi K Higuchi C Hirao M Okamoto M. IL-6 negatively regulates osteoblast differentiation through the SHP2/MEK2 and SHP2/Akt2 pathways in vitro. J Bone Miner Metab. (2014) 32:378–92. 10.1007/s00774-013-0514-1 24122251

[34] Peruzzi B Cappariello A Del Fattore A Rucci N De Benedetti F Teti A. c-Src and IL-6 inhibit osteoblast differentiation and integrate IGFBP5 signalling. Nat Commun. (2012) 3:630. 10.1038/ncomms1651 22252554

[35] Liu X Kirschenbaum A Yao S Levine ACBT-V& H The role of the interleukin-6/gp130 signaling pathway in bone metabolism. In: Litwack G, editor. Vitamins and Hormones. London: Academic Press (2006). p. 341–55. 10.1016/S0083-6729(06)74014-6 17027522

[36] Breyer RM Bagdassarian CK Myers SA Breyer MD. Prostanoid receptors: subtypes and signaling. Annu Rev Pharmacol Toxicol. (2001) 41:661–90. 10.1146/annurev.pharmtox.41.1.661 11264472

[37] Park JY Pillinger MH Abramson SB. Prostaglandin E2 synthesis and secretion: the role of PGE2 synthases. Clin Immunol. (2006) 119:229–40. 10.1016/j.clim.2006.01.016 16540375

[38] Morimoto K Shirata N Taketomi Y Tsuchiya S Segi-Nishida E Inazumi T. Prostaglandin E2–EP3 signaling induces inflammatory swelling by mast cell activation. J Immunol. (2014) 192:1130–7. 10.4049/jimmunol.1300290 24342806

[39] Yao C Sakata D Esaki Y Li Y Matsuoka T Kuroiwa K. Prostaglandin E2–EP4 signaling promotes immune inflammation through TH1 cell differentiation and TH17 cell expansion. Nat Med. (2009) 15:633. 10.1038/nm.1968 19465928

[40] Jia X-Y Chang Y Sun X-J Dai X Wei W. The role of prostaglandin E2 receptor signaling of dendritic cells in rheumatoid arthritis. Int Immunopharmacol. (2014) 23:163–9. 10.1016/j.intimp.2014.08.024 25196430

[41] Honda T Segi-Nishida E Miyachi Y Narumiya S Prostacyclin-IP signaling and prostaglandin E_2_-P2/EP4 signaling both mediate joint inflammation in mouse collagen-induced arthritis. J Exp Med. (2006) 203:325–35. 10.1084/jem.20051310 16446378 PMC2118213

[42] Fattahi MJ Mirshafiey A. Prostaglandins and rheumatoid arthritis. Arthritis (2012) 2012:239310. 10.1155/2012/239310 23193470 PMC3502782

[43] Löwik CWGM van der Pluijm G Bloys H Hoekman K Bijvoet OLM Aarden LA. Parathyroid hormone (PTH) and PTH-like protein (PLP) stimulate interleukin-6 production by osteogenic cells: a possible role of interleukin-6 in osteoclastogenesis. Biochem Biophys Res Commun. (1989) 162:1546–1552. 2548501 10.1016/0006-291x(89)90851-6

[44] Greenfield EM Shaw SM Gornik SA Banks MA. Adenyl cyclase and interleukin 6 are downstream effectors of parathyroid hormone resulting in stimulation of bone resorption. J Clin Invest. (1995) 96:1238–44. 10.1172/JCI118157 7657797 PMC185744

[45] Grey A Mitnick M-A Masiukiewicz U Sun B-H Rudikoff S Jilka RL A role for interleukin-6 in parathyroid hormone-induced bone resorption *in Vivo* 1. Endocrinology (1999) 140:4683–90. 10.1210/endo.140.10.7036 10499526

[46] Fabrizio DB Nadia R Andrea DF Barbara P Rita P Maurizio L Impaired skeletal development in interleukin-6–transgenic mice: a model for the impact of chronic inflammation on the growing skeletal system. Arthritis Rheum. (2006) 54:3551–63. 10.1002/art.22175 17075861

[47] Yang X Ricciardi BF Hernandez-Soria A Shi Y Camacho NP Bostrom MPG. Callus mineralization and maturation are delayed during fracture healing in interleukin-6 knockout mice. Bone (2007) 41:928–36. 10.1016/j.bone.2007.07.022 17921078 PMC2673922

[48] Kasahara T Imai S Kojima H Katagi M Kimura H Chan L. Malfunction of bone marrow derived osteoclasts and the delay of bone fracture healing in diabetic mice. Bone (2010) 47:617–25. 10.1016/j.bone.2010.06.014 20601287 PMC2926189

[49] Theis KA Roblin DW Helmick CG Luo R Prevalence and causes of work disability among working-age U.S. adults, 2011–2013, NHIS. Disabil Health J. (2018) 11:108–15. 10.1016/j.dhjo.2017.04.010 PMC11131972 28476583

[50] Verbruggen A De Clerck LS Bridts CH Van Offel JF Stevens WJ. Flow cytometrical determination of interleukin 1β, interleukin 6 and tumour necrosis factor α in monocytes of rheumatoid arthritis patients; relation with parameters of osteoporosis. Cytokine (1999) 11:869–74. 10.1006/cyto.1998.0500 10547275

[51] Hideko N Jian S Masamichi S Keisuke H Tadamitsu K Kazuyuki Y Anti–interleukin-6 receptor antibody therapy reduces vascular endothelial growth factor production in rheumatoid arthritis. Arthritis Rheum. (2003) 48:1521–9. 10.1002/art.11143 12794819

[52] Wong PK Quinn JM Sims NA van Nieuwenhuijze A Campbell IK Wicks IP. Interleukin-6 modulates production of T lymphocyte–derived cytokines in antigen-induced arthritis and drives inflammation-induced osteoclastogenesis. Arthritis Rheum. (2005) 54:158–68. 10.1002/art.21537 16385511

[53] Liang B Song Z Wu B Gardner D Shealy D Song X-Y. Evaluation of anti-IL-6 monoclonal antibody therapy using murine type II collagen-induced arthritis. J Inflamm (Lond) (2009) 6:10. 10.1186/1476-9255-6-10 19368720 PMC2673212

[54] Tanaka K Hashizume M Mihara M Yoshida H Suzuki M Matsumoto Y. Anti-interleukin-6 receptor antibody prevents systemic bone mass loss via reducing the number of osteoclast precursors in bone marrow in a collagen-induced arthritis model. Clin Exp Immunol. (2014) 175:172–80. 10.1111/cei.12201 24028747 PMC3892408

[55] Nowell MA Richards PJ Horiuchi S Yamamoto N Rose-John S Topley N. Soluble IL-6 receptor governs IL-6 activity in experimental arthritis: blockade of arthritis severity by soluble glycoprotein 130. J Immunol. (2003) 171:3202–9. 10.4049/jimmunol.171.6.3202 12960349

[56] de Hooge ASK van de Loo FAJ Bennink MB Arntz OJ de Hooge P van den Berg WB. Male IL-6 gene knock out mice developed more advanced osteoarthritis upon aging. Osteoarthr Cartilage (2005) 13:66–73. 10.1016/j.joca.2004.09.011 15639639

[57] Madhok R Crilly A Watson J Capell HA. Serum interleukin 6 levels in rheumatoid arthritis: correlations with clinical and laboratory indices of disease activity. Ann Rheum Dis. (1993) 52:232–4. 10.1136/ard.52.3.232 8484679 PMC1005024

[58] Clarke T FDA declines to approve J&J arthritis drug sirukumab. Reuters. Washington, DC (2017).

[59] FDA Approves the ACTPen for Genentech's Actemra a Single-Dose Prefilled Autoinjector for the Treatment of Rheumatoid Arthritis Giant Cell Arteritis and Two Forms of Juvenile Arthritis | Business Wire. BusinessWire (2018) Available online at: https://www.businesswire.com/news/home/20181126005067/en/ (Accessed November 30, 2018)

[60] Garnero P Thompson E Woodworth T Smolen JS Rapid and sustained improvement in bone and cartilage turnover markers with the antiâ€“interleukin-6 receptor inhibitor tocilizumab plus methotrexate in rheumatoid arthritis patients with an inadequate response to methotrexate: results from a substudy of the multicenter double-blind, placebo-controlled trial of tocilizumab in inadequate responders to methotrexate alone. Arthritis Rheum. (2010) 62:33–43. 10.1002/art.25053 20039425

[61] Hashimoto J Garnero P van der Heijde D Miyasaka N Yamamoto K Kawai S. Humanized anti-interleukin-6-receptor antibody (tocilizumab) monotherapy is more effective in slowing radiographic progression in patients with rheumatoid arthritis at high baseline risk for structural damage evaluated with levels of biomarkers, radiography, and BMI: data from the SAMURAI study. Mod Rheumatol. (2011) 21:10–5. 10.3109/s10165-010-0325-3 20574648 PMC3036807

[62] Karsdal MA Schett G Emery P Harari O Byrjalsen I Kenwright A. IL-6 receptor inhibition positively modulates bone balance in rheumatoid arthritis patients with an inadequate response to anti-tumor necrosis factor therapy: biochemical marker analysis of bone metabolism in the tocilizumab RADIATE study (NCT00106522). Semin Arthritis Rheum. (2012) 42:131–9. 10.1016/j.semarthrit.2012.01.004 22397953

[63] Kanbe K Nakamura A Inoue Y Hobo K. Osteoprotegerin expression in bone marrow by treatment with tocilizumab in rheumatoid arthritis. Rheumatol Int. (2012) 32:2669–74. 10.1007/s00296-011-2021-9 21789615

[64] Finzel S Rech J Schmidt S Engelke K Englbrecht M Schett G. Interleukin-6 receptor blockade induces limited repair of bone erosions in rheumatoid arthritis: a micro CT study. Ann Rheum Dis. (2013) 72:396–400. 10.1136/annrheumdis-2011-201075 22586162

[65] Yeo L Toellner K-M Salmon M Filer A Buckley CD Raza K. Cytokine mRNA profiling identifies B cells as a major source of RANKL in rheumatoid arthritis. Ann Rheum Dis. (2011) 70:2022–8. 10.1136/ard.2011.153312 21742639 PMC3184241

[66] Gillilan RE Ayers SD Noy N. Structural basis for activation of fatty acid-binding protein 4. J Mol Biol. (2007) 372:1246–60. 10.1016/j.jmb.2007.07.040 17761196 PMC2032018

[67] Lyman M Lieuw V Richardson R Timmer A Stewart C Granger S. A bispecific antibody that targets IL-6 receptor and IL-17A for the potential therapy of patients with autoimmune and inflammatory diseases. J Biol Chem. (2018) 293:9326–34. 10.1074/jbc.M117.818559 29678878 PMC6005441

[68] Maggio M Guralnik JM Longo DL Ferrucci L. Interleukin-6 in aging and chronic disease: a magnificent pathway. J Gerontol A Biol Sci Med Sci. (2006) 61:575–84. 10.1093/gerona/61.6.575 16799139 PMC2645627

[69] Ershler WB Keller ET. Age-associated increased interleukin-6 gene expression, late-life diseases, and frailty. Annu Rev Med. (2000) 51:245–70. 10.1146/annurev.med.51.1.245 10774463

[70] Daynes RA Araneo BA Ershler WB Maloney C Li GZ Ryu SY. Altered regulation of IL-6 production with normal aging. Possible linkage to the age-associated decline in dehydroepiandrosterone and its sulfated derivative. J Immunol. (1993) 150:5219–30. 8515056

[71] Chisato M Kenichiro K Toshihide M Osamu C Yoshiko O Maki A Endogenous bone-resorbing factors in estrogen deficiency: cooperative effects of IL-1 and IL-6. J Bone Miner Res. (2009) 10:1365–73. 10.1002/jbmr.5650100914 7502709

[72] Jilka RL Hangoc G Girasole G Passeri G Williams DC Abrams JS. Increased osteoclast development after estrogen loss: mediation by interleukin-6. Science (1992) 257:88–91. 1621100 10.1126/science.1621100

[73] Kramer PR Kramer SF Guan G 17β-estradiol regulates cytokine release through modulation of CD16 expression in monocytes and monocyte-derived macrophages. Arthritis Rheum. (2004) 50:1967–75. 10.1002/art.20309 15188374

[74] Yasui T Maegawa M Tomita J Miyatani Y Yamada M Uemura H. Changes in serum cytokine concentrations during the menopausal transition. Maturitas (2007) 56:396–403. 10.1016/j.maturitas.2006.11.002 17164077

[75] Karin M. How NF-κB is activated: the role of the IκB kinase (IKK) complex. Oncogene (1999) 18:6867. 10.1038/sj.onc.1203219 10602462

[76] Sun WH Keller ET Stebler BS Ershler WB Estrogen inhibits phorbol ester-induced IκBα transcription and protein degradation. Biochem Biophys Res Commun. (1998) 244:691–5. 10.1006/bbrc.1998.8324 9535726

[77] Poli V Balena R Fattori E Markatos A Yamamoto M Tanaka H. Interleukin-6 deficient mice are protected from bone loss caused by estrogen depletion. EMBO J. (1994) 13:1189–96. 10.1002/j.1460-2075.1994.tb06368.x 8131749 PMC394928

[78] Li X Zhou Z Zhang Y Yang H. IL-6 contributes to the defective osteogenesis of bone marrow stromal cells from the vertebral body of the glucocorticoid-induced osteoporotic mouse. PLoS ONE (2016) 11:e0154677. 10.1371/journal.pone.0154677 27128729 PMC4851291

[79] Roland A Christina B Gerhard K Jochen Z Josef S Georg S Inhibition of interleukin-6 receptor directly blocks osteoclast formation *in vitro* and *in vivo*. Arthritis Rheum. (2009) 60:2747–56. 10.1002/art.24781 19714627

[80] Ferrari SL Garnero P Emond S Montgomery H Humphries SE Greenspan SL. A functional polymorphic variant in the interleukin-6 gene promoter associated with low bone resorption in postmenopausal women. Arthritis Rheum (2001) 44:196–201. 10.1002/1529-0131(200101)44:1<196::AID-ANR26>3.0.CO;2-5 11212160

[81] Bromberg J Wang TC. Inflammation and cancer: IL-6 and STAT3 complete the link. Cancer Cell (2009) 15:79–80. 10.1016/j.ccr.2009.01.009 19185839 PMC3684978

[82] Hara M Nagasaki T Shiga K Takahashi H Takeyama H. High serum levels of interleukin-6 in patients with advanced or metastatic colorectal cancer: the effect on the outcome and the response to chemotherapy plus bevacizumab. Surg Today (2017) 47:483–9. 10.1007/s00595-016-1404-7 27549777

[83] Ma Y Ren Y Dai Z Wu C Ji Y Xu J. IL-6, IL-8 and TNF-α levels correlate with disease stage in breast cancer patients. Adv Clin Exp Med. (2017) 26:421–6. 10.17219/acem/62120. 28791816

[84] Nakashima J Tachibana M Horiguchi Y Oya M Ohigashi T Asakura H. Serum Interleukin 6 as a prognostic factor in patients with prostate cancer. Clin Cancer Res. (2000) 6:2702–6. 10914713

[85] Ludwig H Nachbaur D Fritz E Krainer M Huber H. Interleukin-6 is a prognostic factor in multiple myeloma. Blood (1991) 77:2794–5. 2043775

[86] Franklin RA Liao W Sarkar A Kim M V Bivona MR Liu K. The cellular and molecular origin of tumor-associated macrophages. Science (2014) 344:921–5. 10.1126/science.1252510 24812208 PMC4204732

[87] Van Overmeire E Laoui D Keirsse J Bonelli S Lahmar Q Van Ginderachter JA. STAT of the union: dynamics of distinct tumor-associated macrophage subsets governed by STAT1. Eur J Immunol. (2014) 44:2238–42. 10.1002/eji.201444870 24975396

[88] Kumari N Dwarakanath BS Das A Bhatt AN. Role of interleukin-6 in cancer progression and therapeutic resistance. Tumor Biol. (2016) 37:11553–72. 10.1007/s13277-016-5098-7 27260630

[89] Erreni M Mantovani A Allavena P. Tumor-associated Macrophages (TAM) and inflammation in colorectal cancer. Cancer Microenviron. (2011) 4:141–54. 10.1007/s12307-010-0052-5 21909876 PMC3170420

[90] Wan S Zhao E Kryczek I Vatan L Sadovskaya A Ludema G. Tumor-associated macrophages produce interleukin 6 and signal via STAT3 to promote expansion of human hepatocellular carcinoma stem cells. Gastroenterology (2014) 147:1393–1404. 10.1053/j.gastro.2014.08.039 25181692 PMC4253315

[91] Erez N Truitt M Olson P Hanahan D. Cancer-associated fibroblasts are activated in incipient neoplasia to orchestrate tumor-promoting inflammation in an NF-κB-dependent manner. Cancer Cell (2010) 17:135–47. 10.1016/j.ccr.2009.12.041 20138012

[92] Karin M Greten FR. NF-κB: linking inflammation and immunity to cancer development and progression. Nat Rev Immunol. (2005) 5:749. 10.1038/nri1703 16175180

[93] Zhang Y Tang H Cai J Zhang T Guo J Feng D. Ovarian cancer-associated fibroblasts contribute to epithelial ovarian carcinoma metastasis by promoting angiogenesis, lymphangiogenesis and tumor cell invasion. Cancer Lett. (2011) 303:47–55. 10.1016/j.canlet.2011.01.011 21310528

[94] Wu X Tao P Zhou Q Li J Yu Z Wang X. IL-6 secreted by cancer-associated fibroblasts promotes epithelial-mesenchymal transition and metastasis of gastric cancer via JAK2/STAT3 signaling pathway. Oncotarget (2017) 8:20741–50. 10.18632/oncotarget.15119 28186964 PMC5400541

[95] Nagasaki T Hara M Nakanishi H Takahashi H Sato M Takeyama H. Interleukin-6 released by colon cancer-associated fibroblasts is critical for tumour angiogenesis: anti-interleukin-6 receptor antibody suppressed angiogenesis and inhibited tumour–stroma interaction. Br J Cancer (2014) 110:469–78. 10.1038/bjc.2013.748 24346288 PMC3899773

[96] Tang D Gao J Wang S Ye N Chong Y Huang Y. Cancer-associated fibroblasts promote angiogenesis in gastric cancer through galectin-1 expression. Tumor Biol. (2016) 37:1889–99. 10.1007/s13277-015-3942-9 26323258

[97] Silva EM Mariano VS Pastrez PRA Pinto MC Castro AG Syrjanen KJ. High systemic IL-6 is associated with worse prognosis in patients with non-small cell lung cancer. PLoS ONE (2017) 12:e0181125. 10.1371/journal.pone.0181125 28715437 PMC5513446

[98] Chen M-F Chen P-T Lu MS Lin PY Chen W-C Lee K-D. IL-6 expression predicts treatment response and outcome in squamous cell carcinoma of the esophagus. Mol Cancer (2013) 12:26. 10.1186/1476-4598-12-26 23561329 PMC3667147

[99] Wu C-T Chen M-F Chen W-C Hsieh C-C. The role of IL-6 in the radiation response of prostate cancer. Radiat Oncol. (2013) 8:159. 10.1186/1748-717X-8-159 23806095 PMC3717100

[100] Heikkila K Ebrahim S Lawlor DA. Systematic review of the association between circulating interleukin-6 (IL-6) and cancer. Eur J Cancer (2008) 44:937–45. 10.1016/j.ejca.2008.02.047 18387296

[101] Shintani Y Fujiwara A Kimura T Kawamura T Funaki S Minami M. IL-6 secreted from cancer-associated fibroblasts mediates chemoresistance in NSCLC by increasing epithelial-mesenchymal transition signaling. J Thorac Oncol. (2016) 11:1482–92. 10.1016/j.jtho.2016.05.025 27287412

[102] Guo Y Xu F Lu T Duan Z Zhang Z. Interleukin-6 signaling pathway in targeted therapy for cancer. Cancer Treat Rev. (2012) 38:904–10. 10.1016/j.ctrv.2012.04.007 22651903

[103] Di G-H Liu Y Lu Y Liu J Wu C Duan H-F. IL-6 secreted from senescent mesenchymal stem cells promotes proliferation and migration of breast cancer cells. PLoS ONE (2014) 9:e113572. 10.1371/journal.pone.0113572 25419563 PMC4242635

[104] Sullivan NJ Sasser AK Axel AE Vesuna F Raman V Ramirez N. Interleukin-6 induces an epithelial–mesenchymal transition phenotype in human breast cancer cells. Oncogene (2009) 28:2940–7. 10.1038/onc.2009.180 19581928 PMC5576031

[105] Wei L-H Kuo M-L Chen C-A Chou C-H Lai K-B Lee C-N. Interleukin-6 promotes cervical tumor growth by VEGF-dependent angiogenesis via a STAT3 pathway. Oncogene (2003) 22:1517. 10.1038/sj.onc.1206226 12629515

[106] Adachi Y Aoki C Yoshio-Hoshino N Takayama K Curiel DT Nishimoto N. Interleukin-6 induces both cell growth and VEGF production in malignant mesotheliomas. Int J Cancer (2006) 119:1303–11. 10.1002/ijc.22006 16642474

[107] Iliopoulos D Hirsch HA Struhl K. An epigenetic switch involving NF-κB, Lin28, let-7 microRNA, and IL6 links inflammation to cell transformation. Cell (2009) 139:693–706. 10.1016/j.cell.2009.10.014 19878981 PMC2783826

[108] Iliopoulos D Jaeger SA Hirsch HA Bulyk ML Struhl K. STAT3 activation of miR-21 and miR-181b-1, via PTEN and CYLD, are part of the epigenetic switch linking inflammation to cancer. Mol Cell (2010) 39:493–506. 10.1016/j.molcel.2010.07.023 20797623 PMC2929389

[109] Hendrayani S-F Al-Harbi B Al-Ansari MM Silva G Aboussekhra A. The inflammatory/cancer-related IL-6/STAT3/NF-κB positive feedback loop includes AUF1 and maintains the active state of breast myofibroblasts. Oncotarget (2016) 7:41974–85. 10.18632/oncotarget.9633 27248826 PMC5173109

[110] Brach MA Hass R Sherman ML Gunji H Weichselbaum R Kufe D. Ionizing radiation induces expression and binding activity of the nuclear factor kappa B. J Clin Invest. (1991) 88:691–5. 10.1172/JCI115354 1864978 PMC295415

[111] Veuger SJ Hunter JE Durkacz BW Ionizing radiation-induced NF-κB activation requires PARP-1 function to confer radio-resistance. Oncogene (2009) 28:832–42. 10.1038/onc.2008.439 19060926 PMC2642763

[112] Criswell T Leskov K Miyamoto S Luo G Boothman DA. Transcription factors activated in mammalian cells after clinically relevant doses of ionizing radiation. Oncogene (2003) 22:5813. 10.1038/sj.onc.1206680 12947388

[113] Zeligs KP Neuman MK Annunziata CM. Molecular pathways: the balance between cancer and the immune system challenges the therapeutic specificity of targeting nuclear factor-κB signaling for cancer treatment. Clin Cancer Res. (2016) 22:4302–8. 10.1158/1078-0432.CCR-15-1374 27422962 PMC5010470

[114] Wu Z-H Miyamoto S. Many faces of NF-κB signaling induced by genotoxic stress. J Mol Med. (2007) 85:1187–202. 10.1007/s00109-007-0227-9 17607554

[115] Kozakai N Kikuchi E Hasegawa M Suzuki E Ide H Miyajima A. Enhancement of radiosensitivity by a unique novel NF-κB inhibitor, DHMEQ, in prostate cancer. Br J Cancer (2012) 107:652–7. 10.1038/bjc.2012.321 22805327 PMC3419964

[116] Selimovic D Porzig BBOW El-Khattouti A Badura HE Ahmad M Ghanjati F. Bortezomib/proteasome inhibitor triggers both apoptosis and autophagy-dependent pathways in melanoma cells. Cell Signal (2013) 25:308–18. 10.1016/j.cellsig.2012.10.004 23079083

[117] Dou QP Zonder JA. Overview of proteasome inhibitor-based anti-cancer therapies: perspective on bortezomib and second generation proteasome inhibitors versus future generation inhibitors of ubiquitin-proteasome system. Curr Cancer Drug Targets (2014) 14:517–36. 10.2174/1568009614666140804154511 25092212 PMC4279864

[118] Periyasamy-Thandavan S Jackson WH Samaddar JS Erickson B Barrett JR. Bortezomib blocks the catabolic process of autophagy via a cathepsin-dependent mechanism, affects endoplasmic reticulum stress, and induces caspase-dependent cell death in antiestrogen–sensitive and resistant ER+ breast cancer cells. Autophagy (2010) 6:19–35. 10.4161/auto.6.1.10323 20110775

[119] Yeong-Shiau P Tzyh-Chyuan H Shuang-En C Ann-Lii C Ming-Kuen L Min-Liang K Interleukin-6 is responsible for drug resistance and anti-apoptotic effects in prostatic cancer cells. Prostate (2004) 60:120–9. 10.1002/pros.20057 15162378

[120] Wei L-H Kuo M-L Chen C-A Chou C-H Cheng W-F Chang M-C. The anti-apoptotic role of interleukin-6 in human cervical cancer is mediated by up-regulation of Mcl-1 through a PI 3-K/Akt pathway. Oncogene (2001) 20:5799. 10.1038/sj.onc.1204733 11593385

[121] Farrell ML Reagan MR. Soluble and cell–cell-mediated drivers of proteasome inhibitor resistance in multiple myeloma. Front Endocrinol (Lausanne) (2018) 9:218. 10.3389/fendo.2018.00218 29765356 PMC5938346

[122] Gesto DS Cerqueira NM Fernandes PA Ramos MJ. Gemcitabine: a critical nucleoside for cancer therapy. Curr Med Chem. (2012) 19:1076–87. 10.2174/092986712799320682 22257063

[123] Moran DM Mattocks MA Cahill PA Koniaris LG McKillop IH Interleukin-6 (il-6) mediates g0/g1 growth arrest in hepatocellular carcinoma through a stat 3-dependent pathway. J Surg Res. (2008) 147:23–33. 10.1016/j.jss.2007.04.022 17574577 PMC2587231

[124] Kortylewski M Heinrich PC Mackiewicz A Schniertshauer U Klingmüller U Nakajima K. Interleukin-6 and oncostatin M-induced growth inhibition of human A375 melanoma cells is STAT-dependent and involves upregulation of the cyclin-dependent kinase inhibitor p27/Kip1. Oncogene (1999) 18:3742. 10391682 10.1038/sj.onc.1202708

[125] Bellido T O'Brien CA Roberson PK Manolagas SC. Transcriptional activation of the p21(WAF1,CIP1,SDI1) gene by interleukin-6 type cytokines. A prerequisite for their pro-differentiating and anti-apoptotic effects on human osteoblastic cells. J Biol Chem. (1998) 273:21137–44. 9694869 10.1074/jbc.273.33.21137

[126] Chipoy C Brounais B Trichet V Battaglia S Berreur M Oliver L. Sensitization of osteosarcoma cells to apoptosis by oncostatin M depends on STAT5 and p53. Oncogene (2007) 26:6653. 10.1038/sj.onc.1210492 17471233

[127] Patel LR Camacho DF Shiozawa Y Pienta KJ Taichman RS. Mechanisms of cancer cell metastasis to the bone: a multistep process. Future Oncol. (2011) 7:1285–97. 10.2217/fon.11.112 22044203 PMC3258525

[128] Tiedemann K Hussein O Sadvakassova G Guo Y Siegel PM Komarova SV Breast cancer-derived factors stimulate osteoclastogenesis through the Ca(2+)/protein kinase C and transforming growth factor-β/MAPK signaling pathways. J Biol Chem. (2009) 284:33662–70. 10.1074/jbc.M109.010785 19801662 PMC2785208

[129] Tumber A Hill PA. Breast cancer cells induce osteoclast formation by stimulating host IL-11 production and downregulating granulocyte/macrophage colony-stimulating factor. Int J Cancer (2004) 109:653–60. 10.1002/ijc.20056 14999770

[130] Yoshihiro M Naoya F Kazuo O Takashi T Stimulation of interleukin-11 production from osteoblast-like cells by transforming growth factor-β and tumor cell factors. Int J Cancer (1998) 71:422–8. 10.1002/(sici)1097-0215(19970502)71:3<422::aid-ijc20>3.0.co;2-g 9139879

[131] Goda Ta Shimo S Yoshihama Y Mohammad N Hassan M Ibaragi S. Bone destruction by invading oral squamous carcinoma cells mediated by the transforming growth factor-β signalling pathway. Anticancer Res Int J Cancer Res Treat (2010) 30:2615–23. 20682990

[132] Sohara Y Shimada H DeClerck YA. Mechanisms of bone invasion and metastasis in human neuroblastoma. Cancer Lett. (2005) 228:203–9. 10.1016/j.canlet.2005.01.059 15975706

[133] Galson DL Silbermann R Roodman GD. Mechanisms of multiple myeloma bone disease. Bonekey Rep. (2012) 1:135. 10.1038/bonekey.2012.135 23951515 PMC3727863

[134] Tang C-H Chuang J-Y Fong Y-C Maa M-C Way T-D Hung C-H. Bone-derived SDF-1 stimulates IL-6 release via CXCR4, ERK and NF-κB pathways and promotes osteoclastogenesis in human oral cancer cells. Carcinogenesis (2008) 29:1483–92. 10.1093/carcin/bgn045 18310089 PMC2516485

[135] Gazitt Y Akay C. Mobilization of myeloma cells involves SDF-1/CXCR4 signaling and downregulation of VLA-4. Stem Cells (2008) 22:65–73. 10.1634/stemcells.22-1-65 14688392

[136] Aggarwal R Ghobrial IM Roodman GD. Chemokines in multiple myeloma. Exp Hematol. (2006) 34:1289–95. 10.1016/j.exphem.2006.06.017 16982321 PMC3134145

[137] Aiuti A Webb IJ Bleul C Springer T Gutierrez-Ramos JC. The chemokine SDF-1 is a chemoattractant for human CD34(+) hematopoietic progenitor cells and provides a new mechanism to explain the mobilization of CD34(+) progenitors to peripheral blood. J Exp Med. (1997) 185:111–20. 10.1084/jem.185.1.111 8996247 PMC2196104

[138] Nie Y Waite J Brewer F Sunshine M-J Littman DR Zou Y-R. The role of CXCR4 in maintaining peripheral B cell compartments and humoral immunity. J Exp Med. (2004) 200:1145–56. 10.1084/jem.20041185 15520246 PMC2211858

[139] Dürig J Schmücker U Dührsen U. Differential expression of chemokine receptors in B cell malignancies. Leukemia (2001) 15:752–6. 10.1038/sj.leu.2402107 11368435

[140] Moharita AL Taborga M Corcoran KE Bryan M Patel PS Rameshwar P SDF-1a regulation in breast cancer cells contacting bone marrow stroma is critical for normal hematopoiesis. Blood (2006) 108:3245–52. 10.1182/blood-2006-01-017459 16857992

[141] Bi J Li P Li C He J Wang Y Zhang H. The SDF-1/CXCR4 chemokine axis in uveal melanoma cell proliferation and migration. Tumor Biol. (2016) 37:4175–82. 10.1007/s13277-015-4259-4 26490988

[142] Sanz-Rodriguez F Hidalgo A Teixidó J. Chemokine stromal cell-derived factor-1α modulates VLA-4 integrin-mediated multiple myeloma cell adhesion to CS-1/fibronectin and VCAM-1. Blood (2001) 97:346–51. 10.1182/blood.V97.2.346 11154207

[143] Peled A Kollet O Ponomaryov T Petit I Franitza S Grabovsky V. The chemokine SDF-1 activates the integrins LFA-1, VLA-4, and VLA-5 on immature human CD34(+) cells: role in transendothelial/stromal migration and engraftment of NOD/SCID mice. Blood (2000) 95:3289–96. 10828007

[144] Wright N de Lera TL García-Moruja C Lillo R García-Sánchez F Caruz A. Transforming growth factor-β1 down-regulates expression of chemokine stromal cell–derived factor-1: functional consequences in cell migration and adhesion. Blood (2003) 102:1978–84. 10.1182/blood-2002-10-3190 12775566

[145] Azab AK Runnels JM Pitsillides C Moreau A-S Azab F Leleu X. CXCR4 inhibitor AMD3100 disrupts the interaction of multiple myeloma cells with the bone marrow microenvironment and enhances their sensitivity to therapy. Blood (2009) 113:4341–51. 10.1182/blood-2008-10-186668 19139079 PMC2676090

[146] Van de Broek I Leleu X Schots R Facon T Vanderkerken K Van Camp B. Clinical significance of chemokine receptor (CCR1, CCR2 and CXCR4) expression in human myeloma cells: the association with disease activity and survival. Haematologica (2006) 91:200–6. 16461304

[147] Möller C Strömberg T Juremalm M Nilsson K Nilsson G. Expression and function of chemokine receptors in human multiple myeloma. Leukemia (2003) 17:203–10. 10.1038/sj.leu.2402717 12529679

[148] Broek I Vande Asosingh K Vanderkerken K Straetmans N Van Camp B Van Riet I Chemokine receptor CCR2 is expressed by human multiple myeloma cells and mediates migration to bone marrow stromal cell-produced monocyte chemotactic proteins MCP-1,-2 and-3. Br J Cancer (2003) 88:855–62. 10.1038/sj.bjc.6600833 12644822 PMC2377079

[149] Nakayama T Hieshima K Izawa D Tatsumi Y Kanamaru A Yoshie O. Cutting edge: profile of chemokine receptor expression on human plasma cells accounts for their efficient recruitment to target tissues. J Immunol. (2003) 170:1136–40. 10.4049/jimmunol.170.3.1136 12538668

[150] Ohshima S Saeki Y Mima T Sasai M Nishioka K Nomura S. Interleukin 6 plays a key role in the development of antigen-induced arthritis. Proc Natl Acad Sci USA. (1998) 95:8222–6. 10.1073/pnas.95.14.8222 9653168 PMC20957

[151] Loetscher P Seitz M Baggiolini M Moser B. Interleukin-2 regulates CC chemokine receptor expression and chemotactic responsiveness in T lymphocytes. J Exp Med. (1996) 184:569–77. 10.1084/jem.184.2.569 8760810 PMC2192704

[152] Terpos E Christoulas D Katodritou E Bratengeier C Gkotzamanidou M Michalis E. Elevated circulating sclerostin correlates with advanced disease features and abnormal bone remodeling in symptomatic myeloma: reduction post-bortezomib monotherapy. Int J cancer (2012) 131:1466–71. 10.1002/ijc.27342 22052418

[153] McDonald MM Reagan MR Youlten SE Mohanty ST Seckinger A Terry RL. Inhibiting the osteocyte-specific protein sclerostin increases bone mass and fracture resistance in multiple myeloma. Blood (2017) 129:3452–64. 10.1182/blood-2017-03-773341 28515094 PMC5492093

[154] Eda H Santo L Wein MN Hu DZ Cirstea DD Nemani N. Regulation of sclerostin expression in multiple myeloma by Dkk-1; a potential therapeutic strategy for myeloma bone disease. J Bone Miner Res. (2016) 31:1225–34. 10.1002/jbmr.2789 26763740 PMC5002355

[155] Falank C Fairfield H Reagan MR. Reflections on cancer in the bone marrow: adverse roles of adipocytes. Curr Mol Biol Rep. (2017) 3:254–62. 10.1007/s40610-017-0074-6 29399440 PMC5791905

[156] Fairfield H Falank C Harris E Demambro V McDonald M Pettitt JAJ. The skeletal cell-derived molecule sclerostin drives bone marrow adipogenesis. J Cell Physiol. (2017) 233:1156–67. 10.1002/jcp.25976 28460416 PMC5664178

[157] Gavriatopoulou M Dimopoulos M-A Christoulas D Migkou M Iakovaki M Gkotzamanidou M. Dickkopf-1: a suitable target for the management of myeloma bone disease. Expert Opin Ther Targets (2009) 13:839–48. 10.1517/14728220903025770 19530987

[158] Tian E Zhan F Walker R Rasmussen E Ma Y Barlogie B. The role of the Wnt-signaling antagonist DKK1 in the development of osteolytic lesions in multiple myeloma. N Engl J Med. (2003) 349:2483–94. 10.1056/NEJMoa030847 14695408

[159] Florio M Gunasekaran K Stolina M Li X Liu L Tipton B. A bispecific antibody targeting sclerostin and DKK-1 promotes bone mass accrual and fracture repair. Nat Commun. (2016) 7:11505. 10.1038/ncomms11505 27230681 PMC4894982

[160] Gunn WG Conley A Deininger L Olson SD Prockop DJ Gregory CA. A crosstalk between myeloma cells and marrow stromal cells stimulates production of DKK1 and interleukin-6: a potential role in the development of lytic bone disease and tumor progression in multiple myeloma. Stem Cells (2006) 24:986–91. 10.1634/stemcells.2005-0220 16293576

[161] Yeremenko N Zwerina K Rigter G Pots D Fonseca JE Zwerina J Brief report: tumor necrosis factor and interleukin-6 differentially regulate Dkk-1 in the inflamed arthritic joint. Arthritis Rheumatol. (2015) 67:2071–5. 10.1002/art.39183 25941031

[162] Wu Q Zhou X Huang D JI Y Kang F. IL-6 enhances osteocyte-mediated osteoclastogenesis by promoting JAK2 and RANKL activity *in vitro*. Cell Physiol Biochem. (2017) 41:1360–9. 10.1159/000465455 28278513

[163] Menaa C Reddy SV Kurihara N Maeda H Anderson D Cundy T. Enhanced RANK ligand expression and responsivity of bone marrow cells in Paget's disease of bone. J Clin Invest. (2000) 105:1833–8. 10.1172/JCI9133 10862799 PMC378510

[164] Rozen N Ish-Shalom S Rachmiel A Stein H Lewinson D. Interleukin-6 modulates trabecular and endochondral bone turnover in the nude mouse by stimulating osteoclast differentiation. Bone (2000) 26:469–74. 10.1016/S8756-3282(00)00263-5 10773586

[165] Dankbar B Padró T Leo R Feldmann B Kropff M Mesters RM. Vascular endothelial growth factor and interleukin-6 in paracrine tumor-stromal cell interactions in multiple myeloma. Blood (2000) 95:2630–6. 10753844

[166] Tanaka Y Abe M Hiasa M Oda A Amou H Nakano A. Myeloma Cell-osteoclast interaction enhances angiogenesis together with bone resorption: a role for vascular endothelial cell growth factor and osteopontin. Clin Cancer Res. (2007) 13:816–23. 10.1158/1078-0432.CCR-06-2258 17289872

[167] Podar K Anderson KC. The pathophysiologic role of VEGF in hematologic malignancies: therapeutic implications. Blood (2005) 105:1383–95. 10.1182/blood-2004-07-2909 15471951

[168] Candé C Cohen I Daugas E Ravagnan L Larochette N Zamzami N. Apoptosis-inducing factor (AIF): a novel caspase-independent death effector released from mitochondria. Biochimie (2002) 84:215–22. 10.1016/S0300-9084(02)01374-3 12022952

[169] Lu Z Xu S. ERK1/2 MAP kinases in cell survival and apoptosis. IUBMB Life (2006) 58:621–31. 10.1080/15216540600957438 17085381

[170] Tu Y Renner S Xu F Fleishman A Taylor J Weisz J. BCL-X expression in multiple myeloma: possible indicator of chemoresistance. Cancer Res. (1998) 58:256–62. 9443402

[171] Zhang B Gojo I Fenton RG. Myeloid cell factor−1 is a critical survival factor for multiple myeloma. Blood (2002) 99:1885–93. 10.1182/blood.V99.6.1885 11877256

[172] Voorhees PM Manges RF Sonneveld P Jagannath S Somlo G Krishnan A A phase 2 multicentre study of siltuximab, an anti-interleukin-6 monoclonal antibody, in patients with relapsed or refractory multiple myeloma. Br J Haematol. (2013) 161:357–66. 10.1111/bjh.12266 23432640 PMC5837861

[173] Orlowski RZ Gercheva L Williams C Sutherland H Robak T Masszi T A phase ii, randomized, double-blind, placebo-controlled study of siltuximab (Anti-IL-6 mAb) and bortezomib versus bortezomib alone in patients with relapsed or refractory multiple myeloma. Am J Hematol. (2015) 90:42–9. 10.1002/ajh.23868 25294016 PMC4737504

[174] Derenne S Monia B Dean NM Taylor JK Rapp M-J Harousseau J-L. Antisense strategy shows that Mcl-1 rather than Bcl-2 or Bcl-x(L) is an essential survival protein of human myeloma cells. Blood (2002) 100:194–9. 10.1182/blood.V100.1.194 12070027

[175] Chauhan D Li G Hideshima T Podar K Shringarpure R Mitsiades C. Blockade of ubiquitin-conjugating enzyme CDC34 enhances anti-myeloma activity of Bortezomib/Proteasome inhibitor PS-341. Oncogene (2004) 23:3597. 10.1038/sj.onc.1207458 15094775

[176] Xiang R-F Wang Y Zhang N Xu W-B Cao Y Tong J. MK2206 enhances the cytocidal effects of bufalin in multiple myeloma by inhibiting the AKT/mTOR pathway. Cell Death Dis. (2017) 8:e2776. 10.1038/cddis.2017.188 28492559 PMC5520709

[177] Hodge DR Peng B Cherry JC Hurt EM Fox SD Kelley JA. Interleukin 6 supports the maintenance of p53 tumor suppressor gene promoter methylation. Cancer Res. (2005) 65:4673–82. 10.1158/0008-5472.CAN-04-3589 15930285

[178] Harada T Hideshima T Anderson KC. Histone deacetylase inhibitors in multiple myeloma: from bench to bedside. Int J Hematol. (2016) 104:300–9. 10.1007/s12185-016-2008-0 27099225

[179] He J Chen Q Yang Y Li Y Yang L Huang H The novel subtype-selective histone deacetylase (HDAC) inhibitor, chidamide, exerts dual anti-myeloma and bone protective effect *in vitro* and *in vivo*. Blood (2017) 130:5392.

[180] Okwan-Duodu D Umpierrez GE Brawley OW Diaz R. Obesity-driven inflammation and cancer risk: role of myeloid derived suppressor cells and alternately activated macrophages. Am J Cancer Res. (2013) 3:21–33. 23359288 PMC3555202

[181] Ellulu MS Patimah I Khaza'ai H Rahmat A Abed Y. Obesity and inflammation: the linking mechanism and the complications. Arch Med Sci. (2017) 13:851–63. 10.5114/aoms.2016.58928 28721154 PMC5507106

[182] Doucette CR Horowitz MC Berry R MacDougald OA Anunciado-Koza R Koza RA. A high fat diet increases bone Marrow Adipose Tissue (MAT) but does not alter trabecular or cortical bone mass in C57BL/6J mice. J Cell Physiol. (2015) 230:2032–7. 10.1002/jcp.24954 25663195 PMC4580244

[183] Falank C Fairfield H Farrell M Reagan MR New bone cell type identified as driver of drug resistance in multiple myeloma: the bone marrow adipocyte. Blood (2017) 130 (Suppl. 1):122.

[184] Fairfield H Falank C Avery L Reagan MR. Multiple myeloma in the marrow: pathogenesis and treatments. Ann N Y Acad Sci. (2016) 1364:32–51. 10.1111/nyas.13038 27002787 PMC4806534

[185] Friedman G LJ H. Obesity and multiple myeloma. Cancer Causes Control (1994) 5: :479–83. 7999970 10.1007/BF01694762

[186] Liu Z Xu J He J Liu H Lin P Wan X. Mature adipocytes in bone marrow protect myeloma cells against chemotherapy through autophagy activation. Oncotarget (2015) 6:34329–41. 10.18632/oncotarget.6020 26455377 PMC4741456

[187] Cao D Zhou H Zhao J Jin L Yu W Yan H. PGC-1α integrates glucose metabolism and angiogenesis in multiple myeloma cells by regulating VEGF and GLUT-4. Oncol Rep. (2014) 31:1205–10. 10.3892/or.2014.2974 24402435

[188] Brighton T Harrison SJ Ghez D Weiss BM Kirsch A Magen H A phase 2, randomized, double-blind, placebo-controlled, multicenter study of siltuximab (Anti IL-6 monoclonal antibody) in patients with high-risk smoldering multiple myeloma. Blood (2017) 130:3155. 10.1158/1078-0432.CCR-18-3470 30890552

[189] Shah JJ Feng L Thomas SK Berkova Z Weber DM Wang M. Siltuximab (CNTO 328) with lenalidomide, bortezomib and dexamethasone in newly-diagnosed, previously untreated multiple myeloma: an open-label phase I trial. Blood Cancer J. (2016) 6:e396. 10.1038/bcj.2016.4 26871714 PMC4771967

[190] Rossi J-F Négrier S James ND Kocak I Hawkins R Davis H. A phase I/II study of siltuximab (CNTO 328), an anti-interleukin-6 monoclonal antibody, in metastatic renal cell cancer. Br J Cancer (2010) 103:1154–62. 10.1038/sj.bjc.6605872 20808314 PMC2967052

[191] Chapurlat R Fibrous dysplasia in the adult. Bone Abstr. (2016) 5:e396 10.1530/boneabs.5.CU2.2

[192] Rossi J-F Lu Z-Y Jourdan M Klein B. Interleukin-6 as a therapeutic target. Clin Cancer Res. (2015) 21:1248–57. 10.1158/1078-0432.CCR-14-2291 25589616

